# Disentangling global and local ring currents†

**DOI:** 10.1039/d2sc05923a

**Published:** 2023-01-16

**Authors:** David Bradley, Michael Jirásek, Harry L. Anderson, Martin D. Peeks

**Affiliations:** a School of Chemistry, University of New South Wales Sydney NSW 2052 Australia m.peeks@unsw.edu.au; b School of Chemistry, University of Glasgow Glasgow G12 8QQ UK; c Department of Chemistry, University of Oxford Oxford OX1 3TA UK

## Abstract

Magnetic field-induced ring currents in aromatic and antiaromatic molecules cause characteristic shielding and deshielding effects in the molecules' NMR spectra. However, it is difficult to analyze (anti)aromaticity directly from experimental NMR data if a molecule has multiple ring current pathways. Here we present a method for using the Biot-Savart law to deconvolute the contributions of different ring currents to the experimental NMR spectra of polycyclic compounds. This method accurately quantifies local and global ring current susceptibilities in porphyrin nanorings, as well as in a bicyclic dithienothiophene-bridged [34]octaphyrin. There is excellent agreement between ring current susceptibilities derived from both experimental and computationally-predicted chemical shifts, and with ring currents calculated by the GIMIC method. Our method can be applied to any polycyclic system, with any number of ring currents, provided that appropriate NMR data are available.

## Abstract 2

Aromatic or antiaromatic ring currents – why not both? We use experimental NMR chemical shifts to identify both local and global ring currents in π-conjugated molecules.

## Graphical abstract

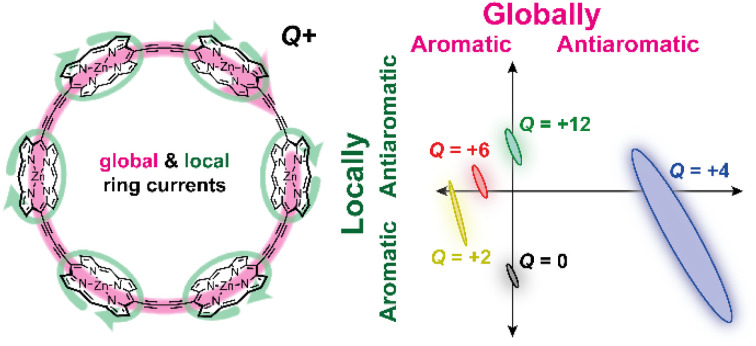

## Introduction

**Fig. 1:**
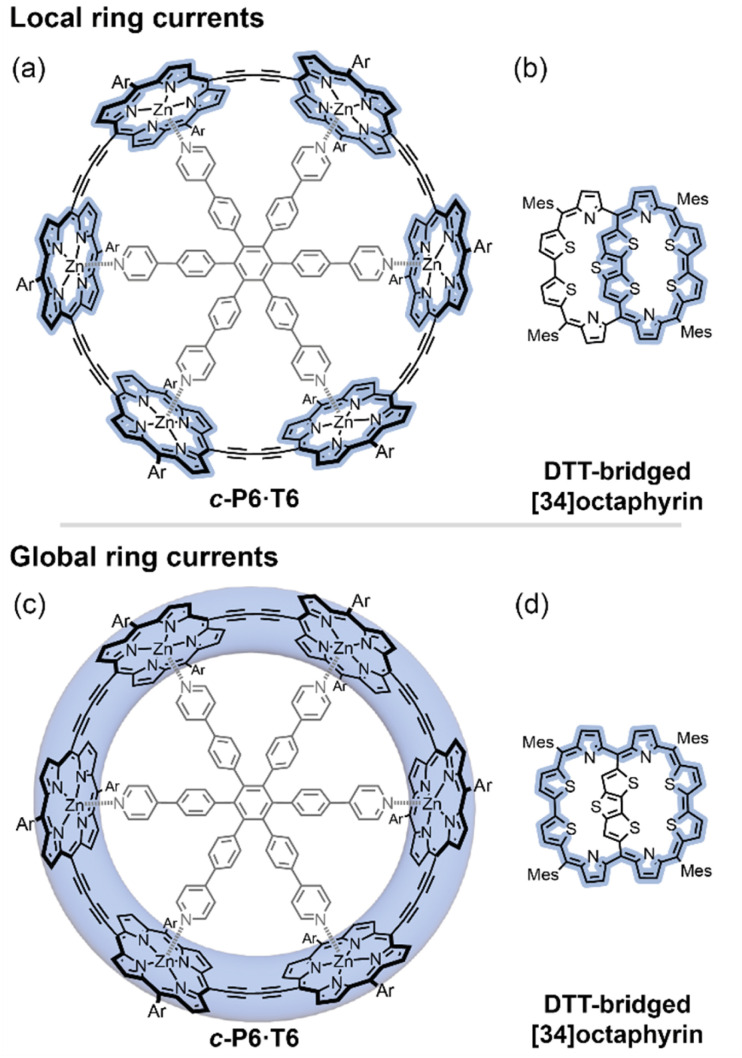
Different local and global ring current paths (blue shading) can be defined in some molecules: a six-porphyrin nanoring *c*-P6·T6 (a and c), and a DTT-bridged [34]octaphyrin (b and d).

Despite being one of the most enduring concepts in chemistry,^1,2^ the assignment of aromaticity in novel molecules remains rife with controversy.^3,4^ Most recent assignments of aromaticity in large molecules have relied heavily on their magnetic properties, which are often easy to measure by NMR spectroscopy, and for which there are relevant computational metrics. These magnetic effects arise, in a classical picture, from a ring current of circulating π-electrons established in response to a magnetic flux through the molecule.^5^ The direction of the ring current is opposite for aromatic and antiaromatic molecules, where it is referred to as diatropic and paratropic, respectively. A ring current induces its own magnetic field, which perturbs the molecule's NMR chemical shifts. Although the ring current model is straightforward to apply to monocyclic neutral annulenes (C_2*n*_H_2*n*_, *e.g.* benzene), the presence of multiple ring current pathways introduces complications. Multiple potential (anti)aromatic circuits can be sketched in most large aromatic molecules (*e.g.*Fig. 1).^1,2,6^ Which of these rings and sub-rings are (anti)aromatic, and can the direction and magnitude of their ring currents be quantified by experiment?

Here we introduce a method for analyzing experimental NMR data which allows us to distinguish the magnetic ring currents of different circuits in large π-conjugated molecules. We apply this method to two systems where both local and global ring current pathways can be drawn: a six-porphyrin nanoring in various oxidation states (*c*-P6·T6*^Q^*, *Q* = 0, +2, +4, +6, +12)^6–8^ and a dithienothiophene (DTT)-bridged [34]octaphyrin (Fig. 1).^9^ We use the NMR data of both systems to disentangle their local and global ring currents, and compare the results to predictions from computational chemistry.

There are several computational methods for quantifying the magnetic criterion of aromaticity. Such methods are extremely valuable in providing insight that often surpasses that accessible by experiment, provided that a suitable level of theory is employed. The challenge of this latter point should not be understated: the choice of DFT functional can dramatically affect the magnitude (and even the direction^8^) of calculated ring currents.^10,11^ The most widely used method for assigning magnetic aromaticity is the nucleus independent chemical shift (NICS), popularized by Schleyer, which calculates the magnetic (de)shielding at points in space within or around a molecule.^12^ NICS can give information about (anti)aromaticity in complex molecules,^13^ and has recently been used to predict the magnitude of ring currents in polycyclic aromatic compounds and porphyrin nanorings.^10,14^

The earliest quantum mechanical methods to predict ring currents were reported in the late 1950s,^15,16^ and several further methods and approaches have since been developed.^17^ The gauge-including magnetically-induced currents (GIMIC) method allows the integration of current density through bonds in a molecule in the presence of a magnetic field, and has been used to quantify global ring currents in porphyrin nanorings.^17–21^ GIMIC has also been used to quantify ring currents in zinc porphyrin nanoshells,^22^ norcorrole dimers,^23^ and in molecules with bicyclic aromatic pathways.^24^ Finally, the Ampère–Maxwell law has been applied to the results of magnetic shielding calculations on symmetric molecules to recover ring current susceptibilities.^25^

In the experimental realm, several authors have previously shown how NMR data can be interpreted using the Biot-Savart law to extract ring current susceptibilities (*I*/*B*, *i.e.* the ratio of the ring current to the external magnetic field strength, in nA T^−1^) for a single current loop.^6,26,27^ Here the (anti)aromatic π-system is treated as a wire loop through which the ring current flows, and the Biot-Savart law is used to calculate the strength of the induced magnetic field at points around this loop corresponding to the locations of NMR-accessible probe nuclei. This approach requires:

(i) A set of chemical shift differences for the probe nuclei (Δ*δ* = *δ*_sample_ − *δ*_ref_, ppm, referred to as the “ring current chemical shift” (RCCS) by Haddon)^26^ where *δ*_ref_ is the chemical shift of the same resonance in the absence of the ring current (ESI Fig. S1†).

(ii) A defined ring current pathway through the molecular structure. This current pathway is used to assign a sensitivity factor known as a ring current geometric factor (RCGF) to each probe nucleus.^6,26^

In ideal cases, the relationship between RCGFs (in μT nA^−1^) and chemical shift differences (Δ*δ*, in ppm) is linear and the slope of the line is the ring current susceptibility (*I*/*B* in nA T^−1^):

1 Δ*δ* = *I*/*B*·RCGF

Some of the present authors recently used this equation to quantify global ring currents in a series of porphyrin nanorings, as function of ring size and oxidation state, treating the molecules as containing only a single macrocyclic ring current circuit.^6^ The experimental NMR data for many π-conjugated macrocycles fit well to the simple linear relationship in eqn (1),^6,11,26^ although Monaco and coworkers have suggested that this model may overlook differences in local ring currents between the macrocycle in question and the reference compound used to estimate *δ*_ref_.^28^ Recently, Matito and coworkers have argued that some of the NMR shifts that we have attributed to global (anti)aromatic ring currents in porphyrin nanorings could arise from local porphyrin ring currents.^3^ While we disagreed with this conclusion on both computational and experimental grounds,^11^ the debate raises a valuable question: When local and global aromatic circuits co-exist in a molecule, how can we determine their relative magnitudes? Here, we present a method for quantifying both local and global ring currents from experimental or calculated NMR data and we benchmark our method with GIMIC calculations, demonstrating that ring current shielding effects in complex, polycyclic molecules can be reliably deconvoluted into both local and global ring current susceptibilities.

## Methods

In our multiple current loop (MCL) model, we expand eqn (1) to include the contributions of multiple ring currents to the observed change in chemical shift Δ*δ*. Each ring current pathway in a complex molecule is associated with its own set of RCGFs. In the molecules studied here, the inclusion of both local and global currents reduces the need for an intercept term in the fit (see ESI Section S7† for discussion). The success of our method relies on the RCGFs associated with each ring current path not being linearly dependent (see ESI Section S3† for more discussion and diagnostics). The MCL method is written, in general form, as:2
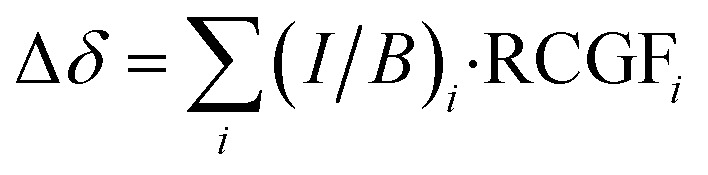


We studied both porphyrin nanorings (*c*-P6·T6*^Q^*) and the DTT-bridged [34]octaphyrin (Fig. 1) using this method. Extensive experimental NMR data are available for both compounds.^6–9^ All of our DFT calculations were performed using Gaussian16,^29^ and molecular structures were optimized at the BLYP35/6-31G* level,^30–34^ in accordance with our previous studies showing that this combination accurately predicts the ^1^H NMR spectra of porphyrin nanorings.^11,34,35^ NMR chemical shifts were calculated at the same level of theory using a solvent model (polarizable continuum model, PCM, with dichloromethane (*c*-P6·T6*^Q^*) or tetrahydrofuran (DTT-[34]octaphyrin)). For the DTT-bridged [34]octaphyrin, we also calculated NMR chemical shifts at the B3LYP/def2-TZVP level for comparison with the literature, using the published geometry.^24^ The calculations on both series used (closed shell) singlet multiplicity, except that *c*-P6·T6^2+^ was treated as the open-shell (broken symmetry) singlet since we previously found that this multiplicity better reproduces experimental NMR chemical shifts.^11^

For calculations with GIMIC, the porphyrin nanorings *c*-P6·T6 were oriented with the *xy* plane defined as the mean plane of the six zinc atoms, leaving the *z*-axis pointing through the central benzene of the T6 template. Global ring currents were calculated by placing a magnetic field along the +*z* axis, whereas local ring currents were calculated for each porphyrin subunit in turn, using a magnetic field parallel to the vector from the center of the nanoring to the center of the porphyrin (approximately in the *xy* plane). The DTT-bridged [34]octaphyrin was oriented with the mean plane of the “big” ring (referred to as “global” in Fig. 1) defined as the *xy* plane, and the magnetic field in GIMIC calculations was applied parallel to the *z* axis.

In a GIMIC calculation, the current through each bond is measured by bisecting it with an integration plane. The size of this plane was optimized as described in ESI Section S4,† to capture as much meaningful current as possible while minimizing spurious contributions from nearby bonds. For *c*-P6·T6*^Q^*, the global ring current is given by the average current through the six central bonds of the butadiyne linkers. The local ring currents for each porphyrin unit were estimated from the average of the integrated currents through each C_α_–C_m_ porphyrin bond (*i.e*., 6 × 8 bonds per nanoring, see ESI Fig. S1† for atom labels). We adopt the convention that diatropic (aromatic) ring currents have negative sign, whereas paratropic (antiaromatic) ring currents are positive.

## Results and discussion

### Porphyrin nanorings

Global (anti)aromaticity has been reported in cationic, anionic, and excited states of π-conjugated porphyrin nanorings.^6–8,11,36^ The archetypal porphyrin nanoring is *c*-P6·T6 (Fig. 1a), in which experimental and computational evidence suggest a change from local aromaticity in each porphyrin subunit in the neutral oxidation state, to global aromaticity/antiaromaticity consistent with Hückel's rule in the +2, +4, and +6 oxidation states.^6^ The +12 oxidation state, corresponding to six dicationic porphyrin subunits, has been assigned as locally antiaromatic.^7^ This molecule is an ideal test case for our model, because the local porphyrin and global macrocycle aromatic pathways are easy to identify, and extensive experimental NMR data are available.^6–8^

**Fig. 2:**
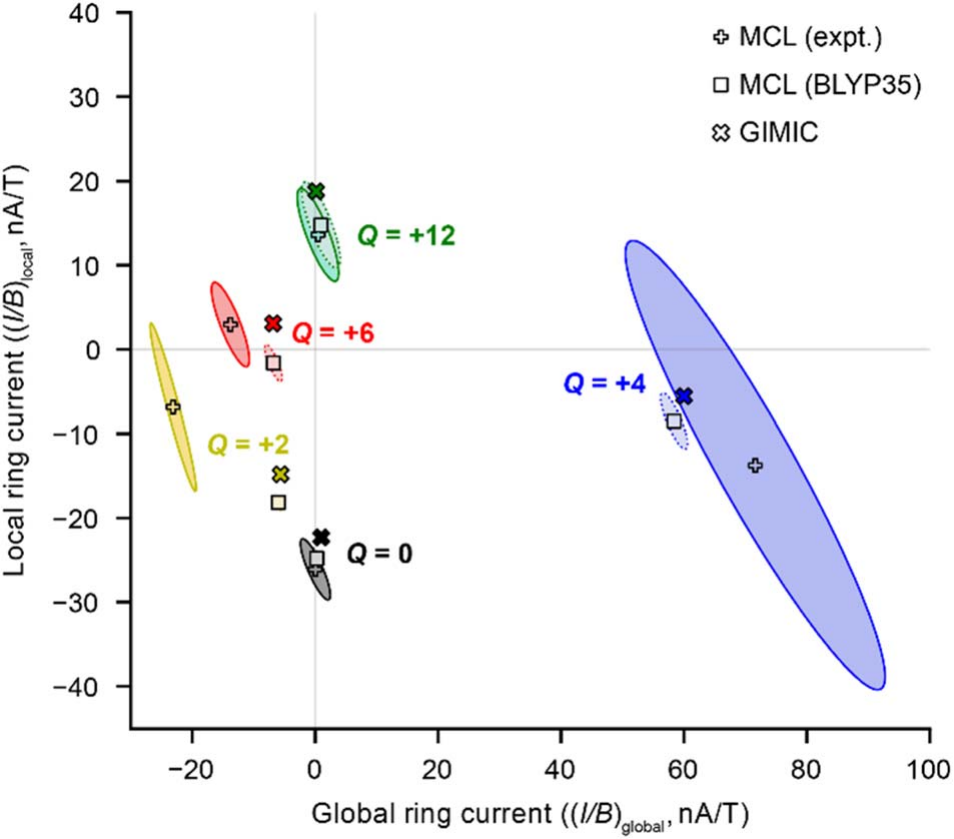
Comparison of local and global ring current susceptibilities estimated using the MCL model for *c*-P6·T6*^Q^* from experimental NMR data (+ symbols), DFT NMR data (squares), and by GIMIC (crosses). The ellipses define the 95% confidence interval for the fit. DFT level of theory: BLYP35/6-31G*. For *Q* = 0 and *Q* = +2, the 95% confidence ellipses from DFT NMR data are hidden by the symbols for the points of best fit.

To quantify the ring current susceptibilities in porphyrin nanorings, we rewrite eqn (2) to include local (porphyrin) and global (macrocycle) ring currents:

3 Δ*δ* = (*I*/*B*)_local_·RCGF_local_ + (*I*/*B*)_global_·RCGF_global_

where (*I*/*B*)_local_ and (*I/B*)_global_ are the local and global ring current susceptibilities in nA T^−1^, and RCGF_local_ and RCGF_global_ quantify the sensitivity of each probe nucleus to local and global ring currents (in μT nA^−1^) flowing through a pathway offset 0.7 Å inside and outside the nanoring, to approximate the π-electron density (see Fig. S21 and ESI Section S1† for discussion). The results of fitting eqn (3) using chemical shift differences (Δ*δ*) from DFT and experiment are shown in Table 1 and Fig. 2. The 95% confidence intervals for the values of the fitted parameters are indicated by ellipses. Fig. 2 also compares the results from the MCL model (eqn (3)) to currents predicted computationally using GIMIC, as discussed below. To quantify the balance between local and global ring currents, we introduce the parameter ‘global ring current fraction’, *f*_global_, which describes the relative magnitude of the global and local (anti)aromatic ring currents. We define this quantity as:

4 *f*_global_ = |(*I*/*B*)_global_|/(|(*I*/*B*)_global_| + |(*I*/*B*)_local_|)

**Table 1:** Comparison of ring current susceptibilities (in nA T^−1^) in *c*-P6·T6*^Q^* (where *Q* = 0, +2, +4, +6, +12) calculated by different methods. GIMIC and DFT results were calculated with BLYP35/6-31G*. RCGFs were calculated from BLYP35-optimized geometries. *f*_global_ is the global ring current fraction as defined by eqn (4)

Method	*I*/*B* (nA T^−1^)	0	+2	+4	+6	+12
MCL (expt.)	(*I*/*B*)_global_	0.0	−23.1	71.6	−13.8	0.4
	(*I*/*B*)_local_	−26.1	−6.8	−13.7	3.0	13.6
	*f*_global_	0.00	0.77	0.84	0.82	0.03
MCL (DFT)	(*I*/*B*)_global_	0.3	−6.0	58.4	−6.8	0.9
	(*I*/*B*)_local_	−24.8	−18.1	−8.5	−1.6	14.8
	*f*_global_	0.01	0.25	0.87	0.81	0.06
GIMIC	(*I*/*B*)_global_	1.0	−5.6	60.0	−6.8	0.2
	(*I*/*B*)_local_^a^	−22.3	−14.8	−5.6	3.1	18.8
	*f*_global_	0.04	0.27	0.91	0.69	0.01

a These values are the mean (*I*/*B*)_local_ averaged over the six porphyrin subunits.

A global ring current fraction *f*_global_ = 0 denotes an NMR spectrum with only local ring current effects, and *f*_global_ = 1 indicates that all of the chemical shift differences (Δ*δ*) can be explained by global (anti)aromaticity. The results are summarized in full in Table 1 and Fig. 2, and show that the *Q* = 0 and *Q* = +12 states evidence only local ring currents, while the spectra of the *Q* = +2, +4, and +6 states evidence both global and local ring current effects.

#### Oxidation states with dominant local (anti)aromaticity

MCL analysis of the *Q* = 0 and +12 oxidation states corroborates the earlier reported analysis,^7^ confirming that *c*-P6·T6 has a negligible global ring current in both of these oxidation states. In the neutral state, the porphyrin subunits are locally aromatic (−26.1 nA T^−1^, Table 1 and Fig. 2), while the 12+ state shows local antiaromaticity (13.6 nA T^−1^). (*I*/*B*)_global_ is so small that the chemical shift differences for the *Q* = 0 and +12 oxidation states fit well to the earlier single current-loop model (eqn (1)) using only RCGF_local_ values (ESI Table S4, Fig. S23 and S27† for the fit using RCGF_global_ values). We performed the same analysis with chemical shift differences predicted by DFT calculations, and as Fig. 2 and Table 1 show, the results are almost identical.

#### Oxidation states with dominant global (anti)aromaticity

Next, we quantified the ring current susceptibilities in the *Q* = +2, +4 and +6 nanorings, which were previously reported to be dominated by global ring currents. The results from the MCL model support our previous assignments – the nanorings exhibit significant global ring currents (Table 1 and Fig. 2). Surprisingly, the MCL model yields higher global ring currents for *Q* = +4 and +6 than our previously-reported results from the single current-loop model (ESI Table S4†).^11^ In addition, each of these globally (anti)aromatic nanorings also feature local porphyrin ring currents. These local currents have opposite signs to the global currents and so partially cancel the latter's effect on the NMR spectra, thus requiring larger values of (*I*/*B*)_global_ to fit the NMR chemical shifts. The values of (*I*/*B*)_global_ (Table 1) are consistent with the presence of global aromatic ring currents in the +2 (−23 nA T^−1^) and +6 (−14 nA T^−1^) states, and a globally antiaromatic ring current in the +4 (72 nA T^−1^) state. For comparison, the ring current susceptibility of benzene, the archetypal aromatic molecule, is −12 nA T^−1^.^6,17,19,22,27,37^

When we used chemical shift differences from DFT (BLYP35) with eqn (3), the signs of the resulting (*I*/*B*)_global_ were consistent with those derived from experimental NMR data, but the magnitudes were smaller, consistent with our previous work.^11^ The difference is especially pronounced for *Q* = +2, for which the experimental (*I*/*B*)_global_ is approximately four times higher than that predicted using DFT chemical shifts. This result probably reflects the uncertainty in the electronic state of the *Q* = +2 state from DFT calculations.^11^

The 95% confidence intervals in Fig. 2 illustrate that most of the uncertainty in the fits is associated with (*I*/*B*)_local_. The sign of (*I*/*B*)_global_, and hence the presence (or absence) of global (anti)aromaticity, can be confidently described. A major cause of uncertainty is that some experimental spectra provide relatively few Δ*δ* values for the fit. In particular, the +4 oxidation state has only four Δ*δ* values. The fits of DFT NMR data have much less uncertainty, because in every case the model includes eight Δ*δ* values.

#### Comparing the MCL model to direct calculation of ring current susceptibilities

**Fig. 3:**
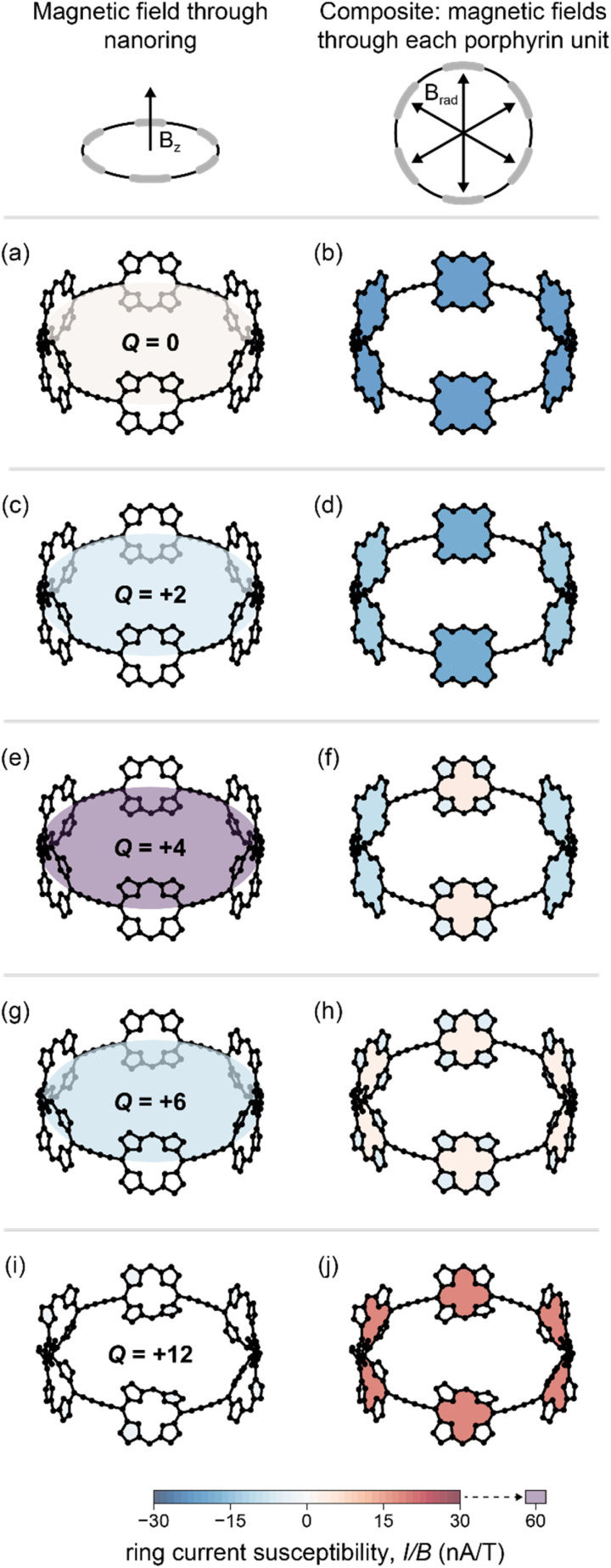
Ring current susceptibilities in *c*-P6·T6*^Q^* for *Q* = 0, +2, +4, +6 and +12, calculated using GIMIC at the BLYP35/6-31G* level. Shaded cycles are colored according to their ring current susceptibility. Left: (*I*/*B*)_global_ induced by a magnetic field along the *z*-axis, perpendicular to the nanoring plane. Right: composite figure showing (*I*/*B*)_local_ induced by a magnetic field through each porphyrin subunit. Red = paratropic currents, blue = diatropic currents. Hydrogen and zinc atoms, T6 template and phenyl groups were removed for clarity.

The MCL model provides an indirect way to quantify ring current susceptibilities from either experimental or calculated NMR data. In the computational realm, the accuracy of this method can be checked by directly measuring the current induced by a magnetic field using GIMIC. If the MCL model is reliable, then we would expect to obtain similar ring current susceptibilities from GIMIC as we do from MCL analysis of DFT chemicals shifts, since both methods are based on the same DFT computational model. The local and global ring current susceptibilities from GIMIC calculations, indicated by crosses in Fig. 2, and listed in Table 1, confirm a high level of consistency with results from the MCL model. A more detailed view of the ring currents from GIMIC is provided by Fig. 3, which shows the global (left) and local (right) ring current susceptibilities for each oxidation state. Figures showing the individual bond currents in these molecules, and discussion of the current pathways, are available in ESI Section S5.†

For the *Q* = 0 and +12 oxidation states, the GIMIC calculations reveal ring current susceptibilities which are negligible for the global pathway (Fig. 3a and i), but significant for the porphyrin subunits (Fig. 3b and j), in keeping with our MCL analysis for both oxidation states.

The global ring current susceptibilities from GIMIC for *Q* = +2, +4, and +6 match extremely well with the values determined using the MCL with DFT chemical shifts (Table 1 and Fig. 2, crosses *vs.* squares), but the local currents show more variability. One explanation for the discrepancy in the (*I/B*)_local_ values may be that, by definition, the MCL model requires that all the porphyrin subunits are identical, whereas GIMIC allows us to examine each individually. According to GIMIC, while all six porphyrin subunits have equivalent antiaromatic ring currents (3.1 nA T^−1^, Fig. 3h) in *c*-P6·T6^6+^, the same is not true for the *Q* = 2+ and 4+ states (Fig. 3d and f). In *c*-P6·T6^2+^, four porphyrins have local ring current susceptibilities of −12.8 nA T^−1^, and the remaining two porphyrins have stronger currents of −18.9 nA T^−1^ (Fig. 3d). The difference is more profound in *c*-P6·T6^4+^, where four porphyrins are locally aromatic (−10.5 nA T^−1^) and two opposing porphyrins are locally antiaromatic (4.3 nA T^−1^, Fig. 3f). This asymmetry is also apparent in the NICS(0)_iso_ grids for both oxidation states (ESI Fig. S28†). This observation might be attributable to these states bearing a non-integer charge per porphyrin. Interestingly, in both *c*-P6·T6^4+^ and *c*-P6·T6^6+^, we can also discern diatropic ring currents (blue shading, Fig. 3f and h) in the pyrrole subunits.

The difference between (*I*/*B*)_local_ values from GIMIC and those from MCL/DFT may also be a consequence of the dimensions of our GIMIC integration planes (ESI Section S4†), or in the choice of *δ*_ref_ for MCL. Nonetheless, the GIMIC results clearly demonstrate that the MCL model performs well at recovering ring current susceptibilities from NMR data. This result is important because it shows that the MCL model is consistent with the widely accepted GIMIC method, when both methods are applied to the same underlying computational model.

**Fig. 4:**
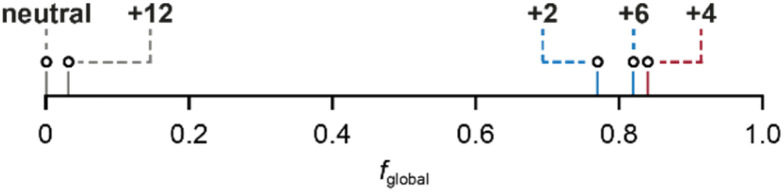
Global ring current fraction, *f*_global_, of *c*-P6·T6*^Q^* (*Q* = 0, +2, +4, +6 and +12), calculated using (*I*/*B*)_global_ and (*I*/*B*)_local_ values from the MCL model using experimental NMR chemical shifts.

Finally, the *f*_global_ (fraction of global ring current) values for all the nanoring oxidation states, from experimental NMR data, are shown in Fig. 4. These values reveal a surprising feature: the spectra of all three oxidation states previously described as globally (anti)aromatic (*Q* = +2, +4, +6) also exhibit approximately the same fraction (∼20%) of local ring current effects.

### DTT-bridged [34]octaphyrin

**Fig. 5:**
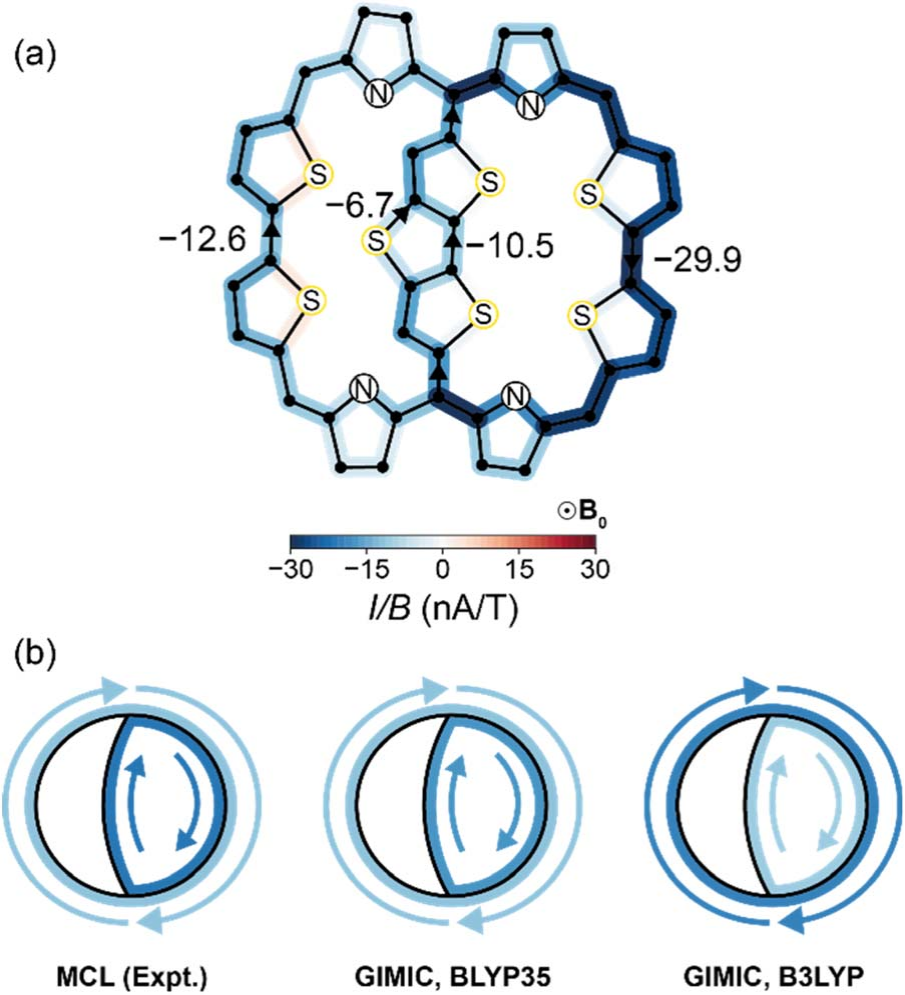
(a) Numerically-integrated currents through bonds in DTT-bridged [34]octaphyrin at the BLYP35/6-31G* level; (b) the relative strengths of small and large ring currents, calculated using eqn (5) (left) and using GIMIC at the BLYP35/6-31G* (middle) and B3LYP/def2-TZVP (right) levels.

Having validated our model on porphyrin nanorings, we turned its application to the DTT-bridged [34]octaphyrin reported by Kim, Chandrashekar, Sessler, Park, Fukuzumi and coworkers.^9^ Sundholm and co-workers reported the results of GIMIC calculations at the B3LYP/def2-TZVP level, which predict ring current susceptibilities of −10.5 nA T^−1^ in the small ring (Fig. 1b) and −22.0 nA T^−1^ in the big ring (Fig. 1d).^24^ We performed our own GIMIC calculations at the BLYP35/6-31G* level (Fig. 5a), which suggested a different assignment: a weaker ring current in the big ring (−12.6 nA T^−1^) than in the small ring (−17.3 nA T^−1^). The effects of the choice of functional can also be observed in the calculated NMR spectra, where BLYP35 chemical shifts are significantly closer to experiment than those from B3LYP (ESI Table S7†).

We then quantified the ring currents using the MCL model (eqn (5)) with experimental NMR data:

5 Δ*δ* = (*I*/*B*)_small_·RCGF_small_ + (*I*/*B*)_big_·RCGF_big_

where the symbols are defined analogously to eqn (3). We calculated RCGFs for the bicyclic compound (see ESI Section S1†) and fitted them to the published experimental NMR data (ESI Tables S6–S8†). The MCL method gives ring current susceptibilities of −22.0 nA T^−1^ and −12.2 nA T^−1^ in the small ring and big ring, respectively, broadly consistent with our GIMIC results calculated using the BLYP35 functional, although inconsistent with the earlier-reported GIMIC//B3LYP results (Fig. 5b). The MCL analysis supports the claim that DTT-bridged [34]octaphyrin has two coexistent ring currents and shows that the MCL method can be used to deconvolute chemical shielding in systems other than porphyrin nanorings. This short study also illustrates the value of experimental NMR data, with the MCL method, in helping to discriminate between inconsistent computational results.

## Conclusions

Large π-conjugated molecules can exhibit multiple ring currents arising from different π-electron pathways. We have shown that a simple model based on the Biot-Savart law can be used to disentangle effects of these multiple ring currents on experimental NMR data. Our MCL model reveals that oxidized porphyrin nanorings, *c*-P6·T6*^Q^* (*Q* = +2, +4 and +6) exhibit both local and global ring currents, the latter consistent with Hückel's rule and at least as strong as the ring current in benzene. For *Q* = 0 and +12, no significant global ring currents are observed and the NMR chemical shift differences are fully described by local porphyrin-based ring currents. We also applied our method to a bicyclic [34]octaphyrin using experimental ^1^H NMR data. Our results indicate that this molecule sustains two overlapping aromatic pathways, and the reported experimental NMR data allow us to assign the relative strengths of each current path. In contrast, DFT calculations give inconsistent estimates of the relative ring current susceptibilities between B3LYP and BLYP35. Our method requires clear NMR assignments of suitably located atoms. In the absence of such data, the method recently reported by Paenurk and Gershoni-Poranne is likely to be more applicable, because it uses the Biot-Savart rule to extract bond currents from NICS values, which can be obtained for arbitrary positions around a molecule.^14^ Our approach is, to the best of our knowledge, unique in its ability to use experimental NMR chemical shifts to quantify the strengths of multiple ring current pathways in complex organic molecules.

## Data availability

The Cartesian coordinates of molecules which we optimized for this paper are available on FigShare: https://doi.org/10.6084/m9.figshare.21391266. The MATLAB code for calculating RCGFs is available on GitHub at https://github.com/mjirasek/Local_vs_Global_RMC. Other data are available in the ESI.†

## Author contributions

D. B.: conceptualization, methodology, software, investigation, validation, writing – original draft; M. J.: methodology, software, investigation, writing – review & editing; H. L. A.: conceptualization, writing – review & editing; M. D. P.: conceptualization, methodology, resources, data curation, writing – review & editing, supervision, project administration, funding acquisition.

## Conflicts of interest

There are no conflicts to declare.

## Supplementary Material

SC-014-D2SC05923A-s001
