# Supplementary material for: Disentangling global and local ring currents
Source: Chem Sci. 2023 Jan 16;14(7):1762–8. doi: 10.1039/d2sc05923a (PMC9930925; doi:10.1039/d2sc05923a)
Supplement: SC-014-D2SC05923A-s001 [file SC-014-D2SC05923A-s001.pdf]

Electronic Supplementary Material (ESI) for Chemical Science.
This journal is © The Royal Society of Chemistry 2023

Electronic Supplementary Information

Disentangling Global and Local Ring Currents

David Bradley,[a] Michael Jirásek,[b] Harry L. Anderson[c] and Martin D. Peeks*,[a]

[a] School of Chemistry, University of New South Wales, Sydney NSW 2052 Australia

[b] School of Chemistry, University of Glasgow, Glasgow G12 8QQ UK

[c] Department of Chemistry, University of Oxford, Oxford OX1 3TA, UK

* MDP: m.peeks@unsw.edu.au

Contents

S1. Computational methods 1
S2. Choice of ring current pathway for MCL analysis 3
S3. Orthogonality of RCGFs 4
S4. GIMIC: Optimizing the size of the integration plane 6
S5. GIMIC: Ring current pathways 9
S5.1. Ring current pathways in ***c*-P6·T6** 9
S5.2. Ring current pathways in ***c*-P6·T6^{2+}** 11
S5.3. Ring current pathways in ***c*-P6·T6^{4+}** 13
S5.4. Ring current pathways in ***c*-P6·T6^{6+}** 15
S5.4. Ring current pathways in ***c*-P6·T6^{12+}** 17
S6. Supplementary figures and tables 19
S7. Statistical analyses 31
S8. References 33

S1. Computational methods

For our computational analysis of ***c*-P6·T6^{Q}** (Q = 0, +2, +4, +6, +12), the trihexylsilyl solubilizing groups were replaced by hydrogen atoms (Figure S1). NMR chemical shifts were calculated at the BLYP35/6-31G* or CAM-B3LYP/6-31G* (with Grimme's D3 dispersion correction[1]) levels of theory,[2–7] with an implicit solvent model (PCM) to match the reported experimental conditions (dichloromethane[8,9] for porphyrin nanorings, tetrahydrofuran[10] for DTT-bridged [34]octaphyrin). Magnetically induced current densities were calculated using GIMIC,[11–13] and were integrated around each bond (see section S4). We visualized the integrated bond currents by coloring each bond according to its current strength (*i.e.*, ring current susceptibility).

Cartesian coordinates for optimized molecules are available on FigShare: https://doi.org/10.6084/m9.figshare.21391266

Ring current geometric factors (RCGFs) were calculated as described in detail in reference [14] using a MATLAB script, updated for the present work, which can be found in the repository (https://github.com/mjirasek/Local_vs_Global_RMC). In the current study, the ring current model (RCM) was built using the classical Biot-Savart law, approximating the ring current circuit path as infinitely thin wire scaffolds.

[34]Octaphyrin. For the analysis of the bicyclic [34]octaphyrin, two RCMs were constructed, for the global and local circuits, following the conjugated paths as proposed in the original reference.[8] The RCM loops were split into two circuits offset by ± 0.7 Å, above and below the normal plane. This plane was defined by the atoms involved in the circuit in the near vicinity. This model was found in our previous studies to yield the most reliable RCGF values for small molecules and mimics the π-conjugation.

Nanorings. The RCMs for the porphyrin nanorings follow the conjugated paths, offset by ± 0.7 Å, analogously to the model for [34]octaphyrin. An exception for defining the offset direction was implemented for paths over butadiyne linkers. At these sections, the normal vector defined by the local atoms result in unphysical twists (toward out-of-plane conjugation). To keep the RCM describing an in-plane conjugation, the offset direction vector (for paths involving butadiyne carbons) was set as toward and away from the center of the nanoring. For further discussion regarding choosing the paths, see Section **S2**.

The RCGFs (in μT/nA) represent the magnetic field (in μT) induced by a ring current of 1 nA. For the comparison of RCGF with experimental NMR data, the experimental chemical shift needs to be referenced to an analogous "non-aromatic chemical shift", *i.e.* the shift that would be observed in absence of the studied ring current. For the nanorings, all references were used as in our previous studies. For the bicyclic [34]octaphyrin, we used a default value for a non-aromatic olefin of 5.8 ppm for all protons, as no direct reference is available. When we fit the RCGFs to chemical shift differences derived from DFT, we used the calculated chemical shift of cyclohexa-1,3-diene (5.87 ppm, BLYP35/6-31G*) as our non-aromatic reference.

The RCGFs from the MCL model (also called composite RCGFs) are defined as:

$$RCGF_{composite} = \frac{(I/B)_{local}}{(I/B)_{local} + (I/B)_{global}} \cdot RCGF_{local} + \frac{(I/B)_{global}}{(I/B)_{global} + (I/B)_{local}} \cdot RCGF_{global}$$

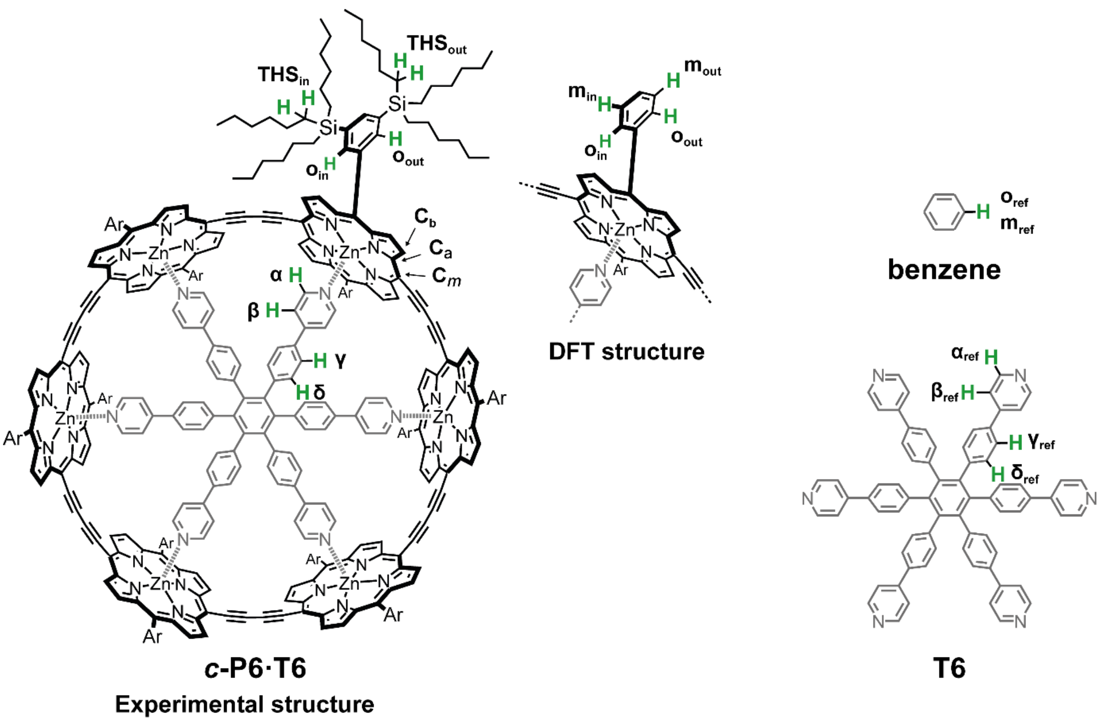

Figure S1: Experimental and DFT model structures of ***c*-P6·T6**, **T6** and benzene in this study.

S2. Choice of ring current pathway for MCL analysis

There are multiple potential current pathways in porphyrin nanorings (Figure S2). The choice of pathway affects RCGF values, so we investigated how equation 2 fits our data for several different models. The global ring current can pass through all four nitrogen atoms of the porphyrin (the **inner path**), or it can pass through the outside of the porphyrin (the **outer path**). The true current pathway is probably a mixture of these options, so the inner and outer pathways can be weighted at 50% each, creating a **mixed path**. The ring current model can also be constructed assuming that current either passes through the π-system, which adds complexity, or through the σ-system, which is simpler to calculate. We compare all variations in Table S1 and S2. There seems to be no significant difference between the results from the σ and π ring current models (compare Tables S1 and S2), suggesting that the simpler σ-model can provide meaningful results. Our method does not include RCGFs arising from pyrrole ring currents.

The choice of ring current pathway (local, global, or mixed) affects I/B_{local} by up to a factor of 2. Consistent with our previous efforts, and to avoid introducing bias into to the model, we used the **mixed** ring current pathway, which is equivalent to the ring current splitting equally at each fork in its path.

Table S1: Ring current susceptibilities for *c*-**P6·T6**$^{6+}$ with different ring current pathways (mix, inner, and outer, defined below). RCGFs were calculated according to the procedure described in S1 – in these data the ring current passes through the π system. See Figure S2 for ring current pathways.

	**Global$_{mix}$**			**Global$_{inner}$**			**Global$_{outer}$**		
	$(I/B)_{global}$ (nA/T)	$(I/B)_{local}$ (nA/T)	RMSE (nA/T)	$(I/B)_{global}$ (nA/T)	$(I/B)_{local}$ (nA/T)	RMSE (nA/T)	$(I/B)_{global}$ (nA/T)	$(I/B)_{local}$ (nA/T)	RMSE (nA/T)
**Local$_{mix}$**	−6.81	−1.62	0.12	−6.91	−1.14	0.14	−6.68	−2.10	0.11
**Local$_{inner}$**	−6.82	−1.84	0.12	−6.92	−1.29	0.14	−6.70	−2.40	0.11
**Local$_{outer}$**	−6.80	−1.44	0.12	−6.90	−1.02	0.14	−6.68	−1.87	0.10

Table S2: Ring current susceptibilities for *c*-**P6·T6**$^{6+}$ with different ring current pathways (mix, inner, and outer, defined below). RCGFs were calculated according to the procedure described in S1 – in these data the ring current passes through the σ system. See Figure S2 for ring current pathways.

	**Global$_{mix}$**			**Global$_{inner}$**			**Global$_{outer}$**		
	$(I/B)_{global}$ (nA/T)	$(I/B)_{local}$ (nA/T)	RMSE (nA/T)	$(I/B)_{global}$ (nA/T)	$(I/B)_{local}$ (nA/T)	RMSE (nA/T)	$(I/B)_{global}$ (nA/T)	$(I/B)_{local}$ (nA/T)	RMSE (nA/T)
**Local$_{mix}$**	−6.82	−1.73	0.12	−6.95	−1.20	0.14	−6.67	−2.26	0.11
**Local$_{inner}$**	−6.82	−1.98	0.12	−6.95	−1.38	0.14	−6.68	−2.59	0.11
**Local$_{outer}$**	−6.82	−1.53	0.12	−6.95	−1.06	0.14	−6.67	−2.00	0.10

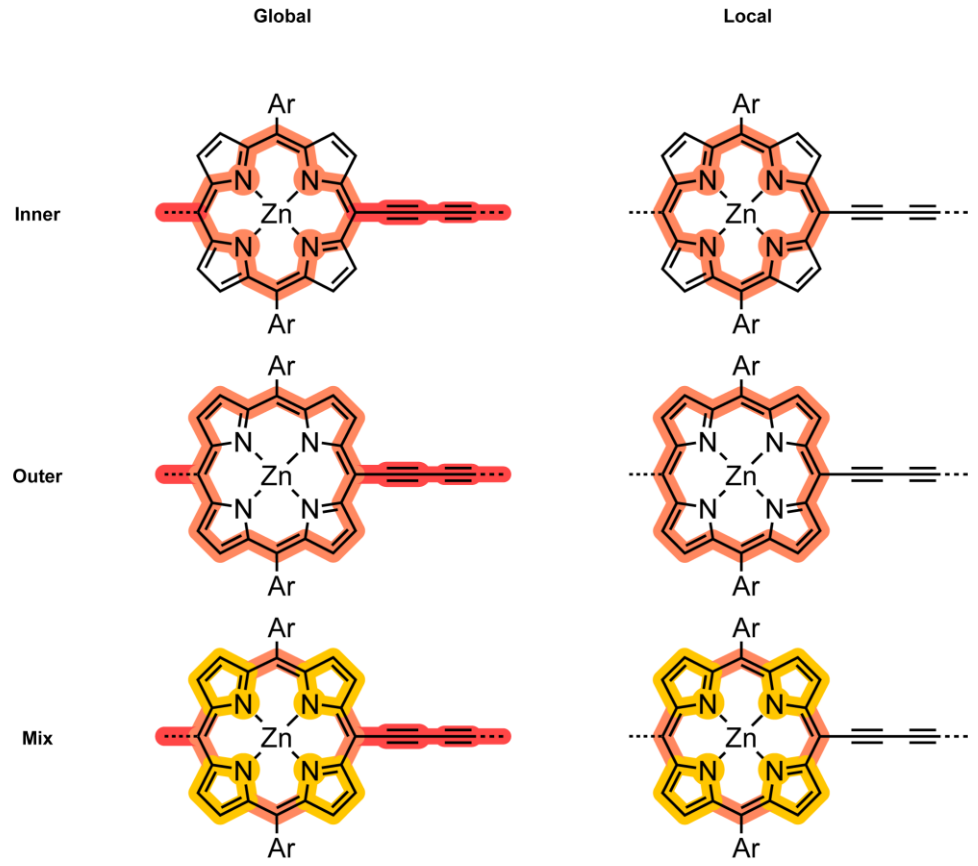

Figure S2: Representative ring current pathways in porphyrin nanorings for the repeating unit of ***c*-P6** as used in Table S1 and Table S2.

S3. Orthogonality of RCGFs

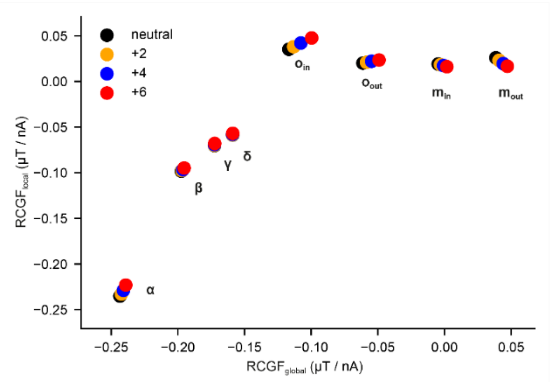

Figure S3: Comparison of $RCGF_{global}$ and $RCGF_{local}$ values calculated for ***c*-P6·T6**^{Q} (Q = 0, +2, +4, +6) for several nuclei. The RCGF values show the sensitivity of each atom to global and local ring currents. If the points for different atoms sit on a straight line, then the local and global currents are indistinguishable by our method.

Accurate quantification of multiple ring currents requires good orthogonality between RCGFs for different circuits. The local and global RCGF values for β, γ, and δ are linearly dependent (Figure S3; a straight line can be drawn between the three points corresponding to these atoms) – these protons alone could not be used to deconvolute the magnetic shielding into local and global ring current components. Other protons, such as $o_{(in)}$, $o_{(out)}$ and α, are not linearly dependent. The $o_{(in)}$ and $o_{(out)}$ protons are sensitive only to shielding arising from global ring currents, whereas including the α proton adds some sensitivity to local ring currents. The fits of different combinations of RCGFs for ***c*-P6·T6**$^{6+}$ are shown in Figure S4.

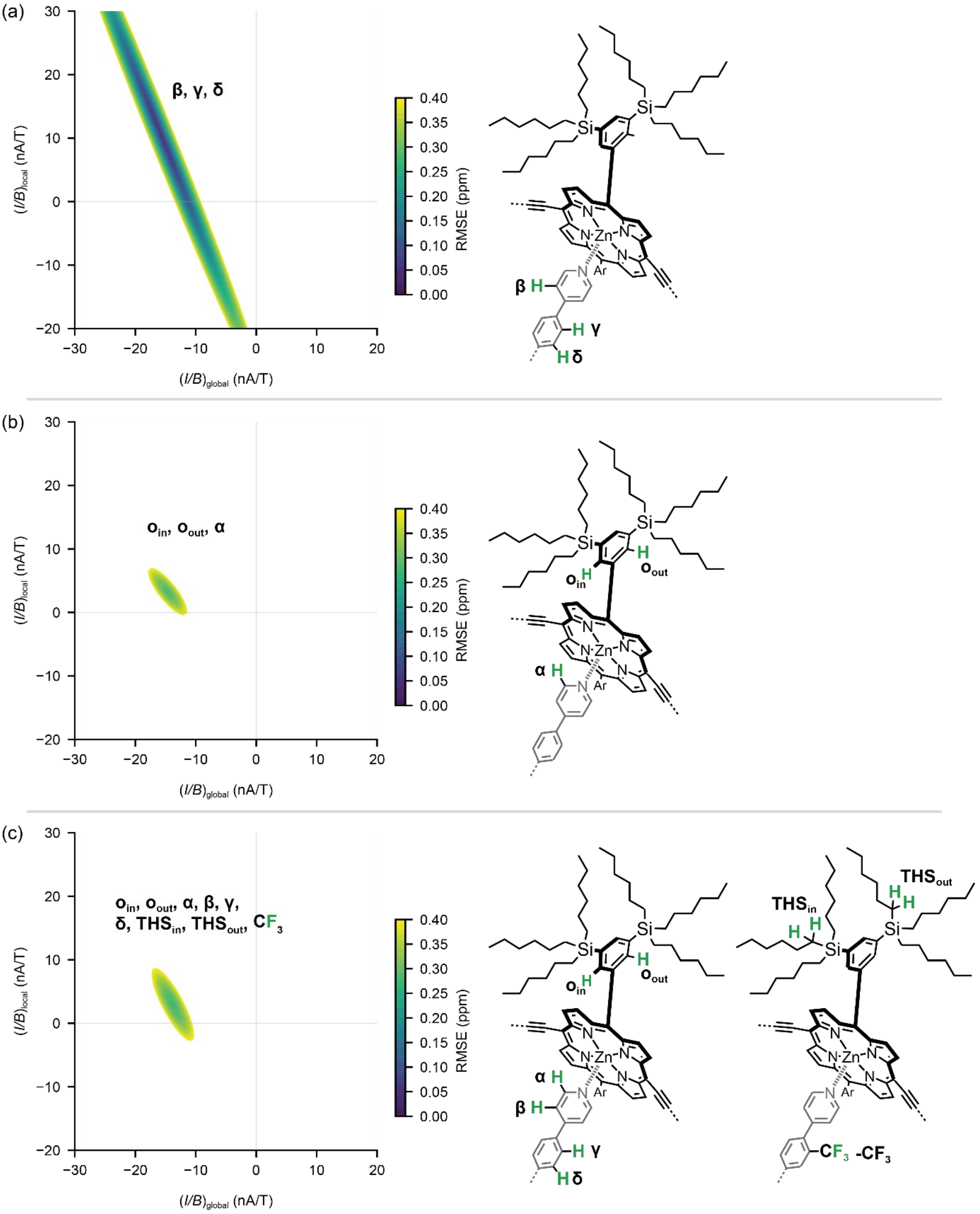

Figure S4: Adequate orthogonality is required for precise measurements of ring current strengths. These plots show the RMSE between experimental and estimated (from equation 3) Δδs for different values of $(I/B)_{global}$ and $(I/B)_{local}$ and different sets of protons, for ***c*-P6·T6**$^{6+}$. (a) The β, γ and δ protons have poor orthogonality, and when only these protons are used in equation 3, the RMSE contour is very imprecise. (b) The $o_{(in)}$, $o_{(out)}$ and α protons have good orthogonality, resulting in a small contour with a small range of potential $(I/B)_{global}$ and $(I/B)_{local}$ values. (c) Including all available protons shows how the fit is similar to that in (b), as a result of good orthogonality.

S4. GIMIC: Optimizing the size of the integration plane

Template-bound porphyrin nanorings are complex structures, and so the extraction of bond-currents from GIMIC calculations requires that the size of the ring current integration plane (red in Fig S6) is chosen carefully. The current integration plane sits perpendicular to the bond axis, and bisects the bond. The plane is required to capture as much of the bond current as possible, without including contamination from other nearby bonds.

We first optimized the width of the integration plane for intra-porphyrin bonds (*i.e.* C_b-C_b, N-C_a, and C_a-C_m in Fig. S5), by extending the plane inside and outside the nanoring, maintaining a constant height of 3.0 Bohr. The integrated ring current strengths do not change significantly for planes wider than 8.0 Bohr, therefore we adopted this plane width for all of our measurements (Fig. S7). We then set out to optimize the height of the integration plane (Figs S8-S9). Increasing the height of the plane for the porphyrin C_b-C_b and N-C_a bonds does not have a clear "best" value. At heights of greater than 4.0 Bohr, which is roughly the center of the pyrrole ring, the integrated current becomes contaminated by the bonds on the opposite side of the pyrrole, and the plane eventually reaches the pyrrole N atom when its height reaches 7.0 Bohr. For the C_a-C_m bonds, the integrated current has a clearer dependence on the height of the integration plane: current strengths increase with plane height from 2.0 – 4.0 Bohr, before decreasing for larger planes. We therefore used a plane with dimensions 8 × 4 Bohr (w × h) for the intra-porphyrin bonds.

The central butadiyne C-C is further from any possible contaminating influences, and so a larger integration plane with dimensions 8 × 8 Bohr could be used (Fig. S10).

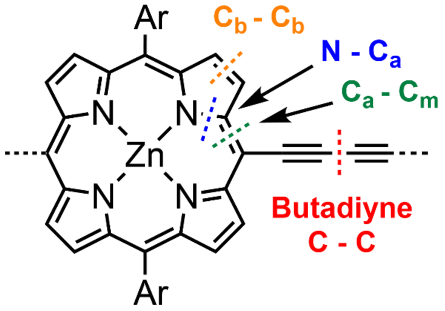

Figure S5: Porphyrin monomer showing the bonds used to optimize the size of the integration plane, as discussed below.

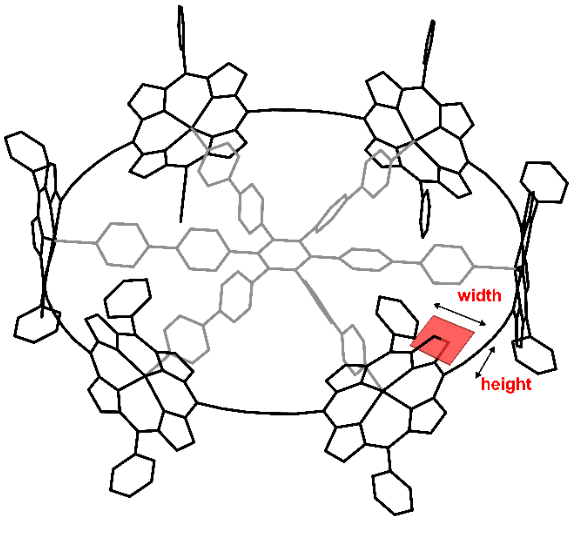

Figure S6: This figure shows an integration plane used to integrate the current across a ***c*-P6·T6** C_b – C_b bond. The height and width of the integration plane was optimized to only integrate the current across the selected bond. Width represents extension of the integration plane towards and away from the centre of the nanoring, height represents extension of the plane away from the plane of the nanoring.

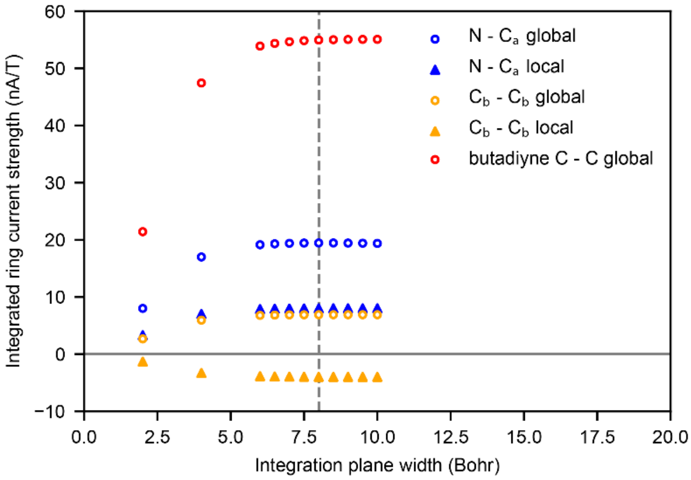

Figure S7: Optimization of the width of the integration plane measuring the ring current induced by a magnetic field along the z axis (global) and the porphyrin radial axis (local) for ***c*-P6·T6**$^{4+}$ (BLYP35/6-31G*). The choice of porphyrin in ***c*-P6·T6**$^{4+}$ affects the results, in this case we select an antiaromatic porphyrin which has aromatic pyrrole rings (hence the difference in sign between N – C_a local and C_b – C_b local. The height of the integration plane is set to 3.0 Bohr. The dashed line indicates the width of the plane which was used in all further GIMIC measurements.

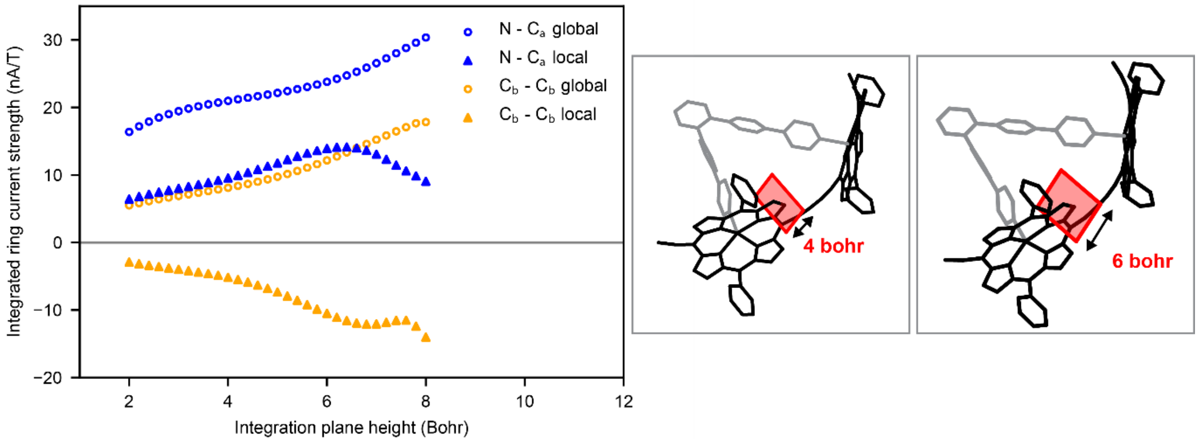

Figure S8: Optimization of the height of the integration plane measuring the ring current induced by a magnetic field along the z axis (global) and the porphyrin radial axis (local) for ***c*-P6·T6**$^{4+}$ (BLYP35/6-31G*). The width of the integration plane is set to 8.0 Bohr. The integration plane becomes contaminated by other sources of ring current at high plane heights, shown on the right is integration planes across the C_b – C_b bond.

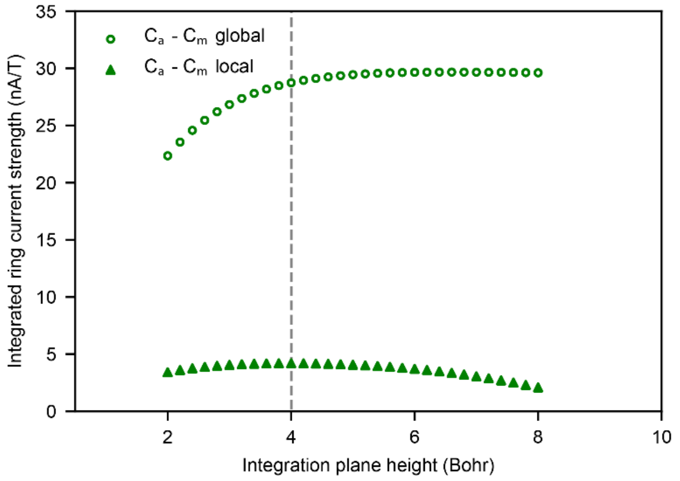

Figure S9: Optimization of the height of the integration plane measuring the ring current induced by a magnetic field along the *z* axis (global) and the porphyrin radial axis (local) for ***c*-P6·T6**$^{4+}$ (BLYP35/6-31G*). The width of the integration plane is set to 8.0 Bohr. The dashed line indicates the height of the plane which was used in all further GIMIC measurements for these bonds.

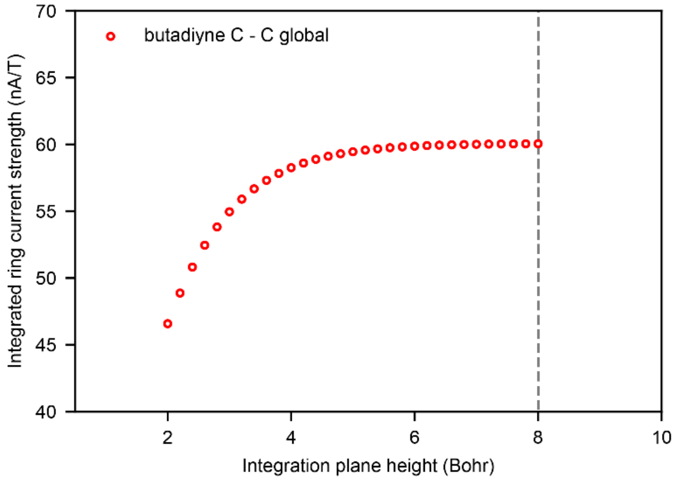

Figure S10: Integration plane height optimization for the inter-porphyrin butadiyne central C – C bond. The width of the integration plane is 8.0 Bohr. This bond is selective for the total global ring current.

S5. GIMIC: Ring current pathways

S5.1. Ring current pathways in *c*-P6·T6

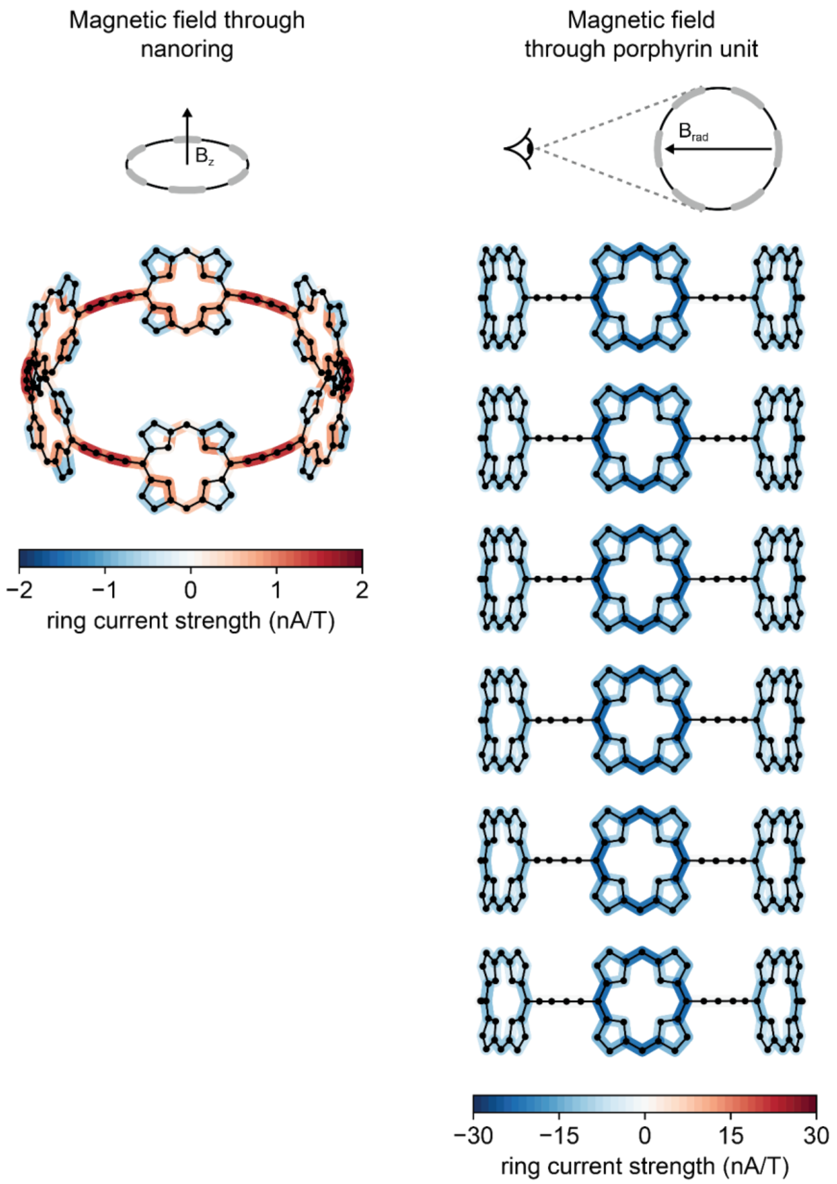

Figure S11: Local ring currents induced in ***c*-P6·T6** by application of a magnetic field along a vector parallel to the radial axis of the nanoring (from the center of the nanoring to the center of a porphyrin), pointing towards the viewer in each subfigure. Each subfigure shows a different porphyrin subunit, though in this oxidation state all are near-identical. Calculated using GIMIC (BLYP35/6-31G*).

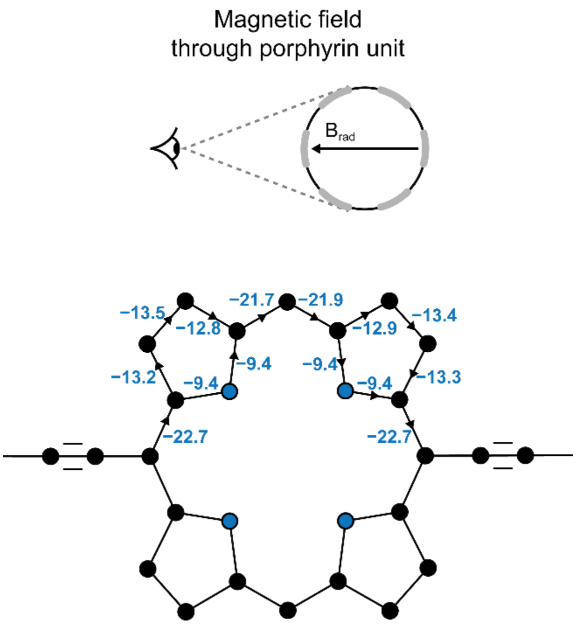

Figure S12: Ring current susceptibilities (in nA/T) calculated using GIMIC for one substituent porphyrin in ***c*-P6·T6**. Clockwise arrows and negative values represent a local diatropic ring current.

The porphyrins in ***c*-P6·T6** sustain a total −22.3 nA/T ring current. This ring current splits unequally at the pyrrole C_a atom, such that ~60% of the current traces the outer 3-bond pathway and the remaining ~40% flows across the pyrrole nitrogen bridge.

S5.2. Ring current pathways in *c*-P6·T6^{2+}

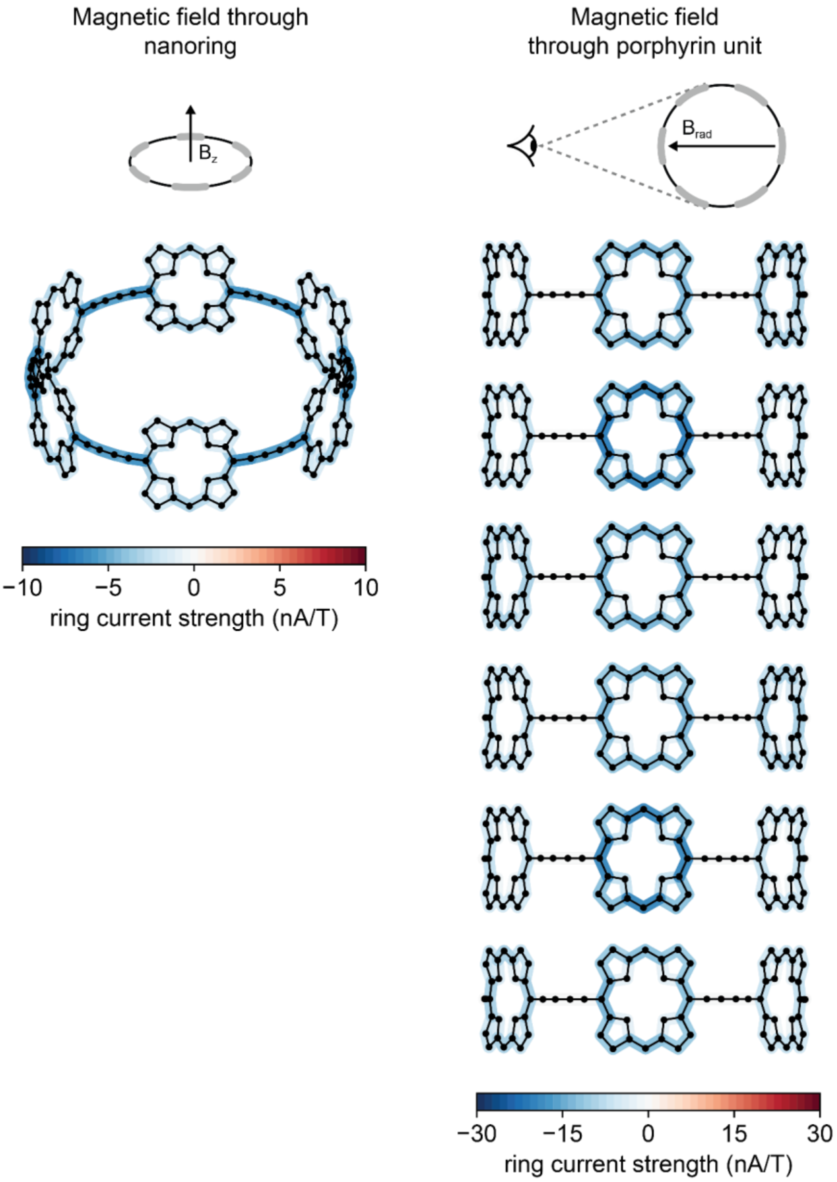

Figure S13: Local ring currents induced in ***c*-P6·T6^{2+}** by application of a magnetic field along a vector parallel to the radial axis of the nanoring (from the center of the nanoring to the center of a porphyrin), pointing towards the viewer in each subfigure. Each subfigure shows a different porphyrin subunit. Calculated using GIMIC (BLYP35/6-31G*).

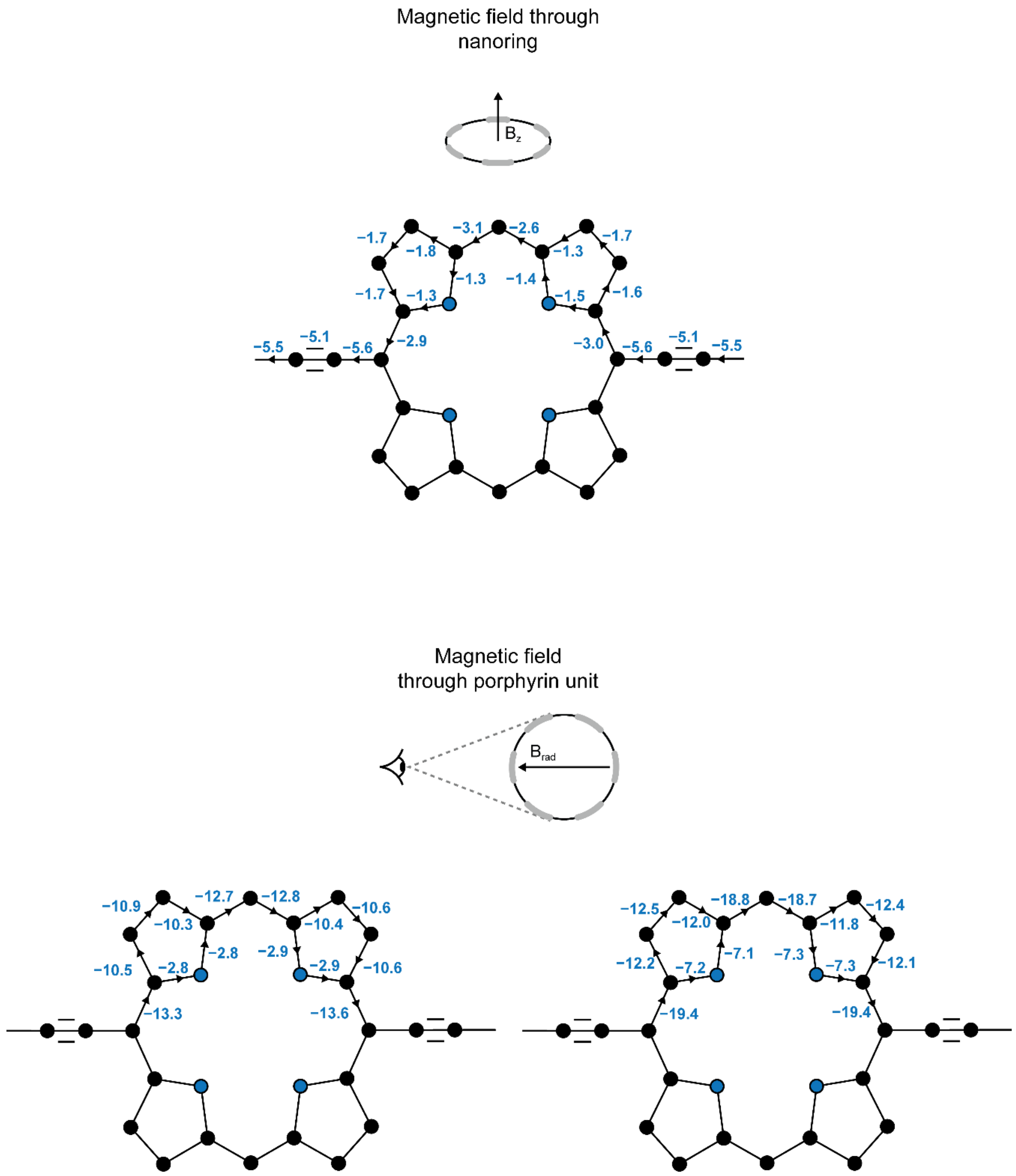

Figure S14: Ring current susceptibilities (in nA/T) calculated using GIMIC for one substituent porphyrin in ***c*-P6·T6**$^{2+}$. The upper figure shows global ring current susceptibilities through a substituent porphyrin. The lower figures show local ring current susceptibilities.

The global ring current in ***c*-P6·T6**$^{2+}$ splits equally across the butadiyne-connected C_a – C_m bonds. The pathway then splits equally again at each pyrrole C_a atom.

There are two different porphyrin environments in ***c*-P6·T6**$^{2+}$: two porphyrins have stronger ring currents of −18.9 nA/T and four porphyrins with −12.8 nA/T ring currents. Their current pathways are also different: in the porphyrins with stronger currents, ~60% of the ring current flows over the outer 3-bond path, while in the porphyrins with weaker currents ~80% flows over this path.

S5.3. Ring current pathways in *c*-P6·T6^{4+}

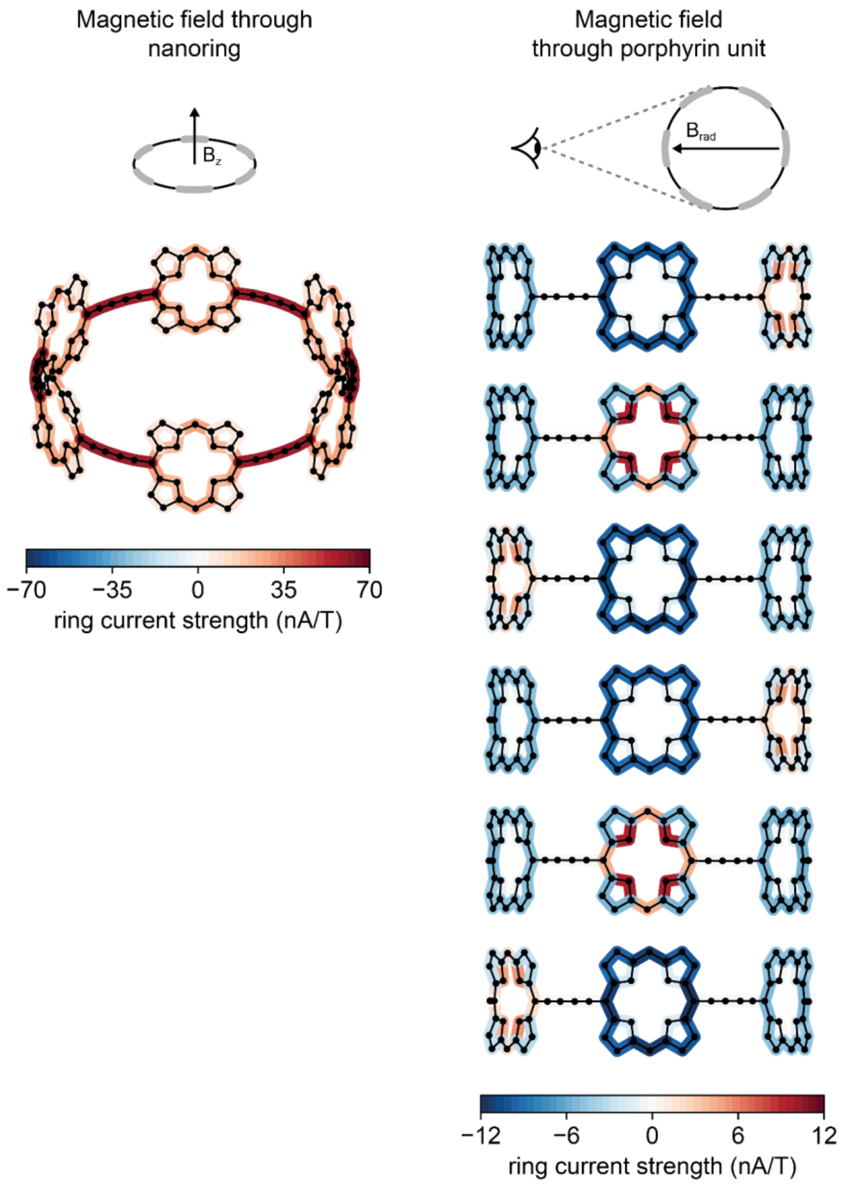

Figure S15: Local ring currents induced in ***c*-P6·T6^{4+}** by application of a magnetic field along a vector parallel to the radial axis of the nanoring (from the center of the nanoring to the center of a porphyrin), pointing towards the viewer in each subfigure. Each subfigure shows a different porphyrin subunit. Calculated using GIMIC (BLYP35/6-31G*).

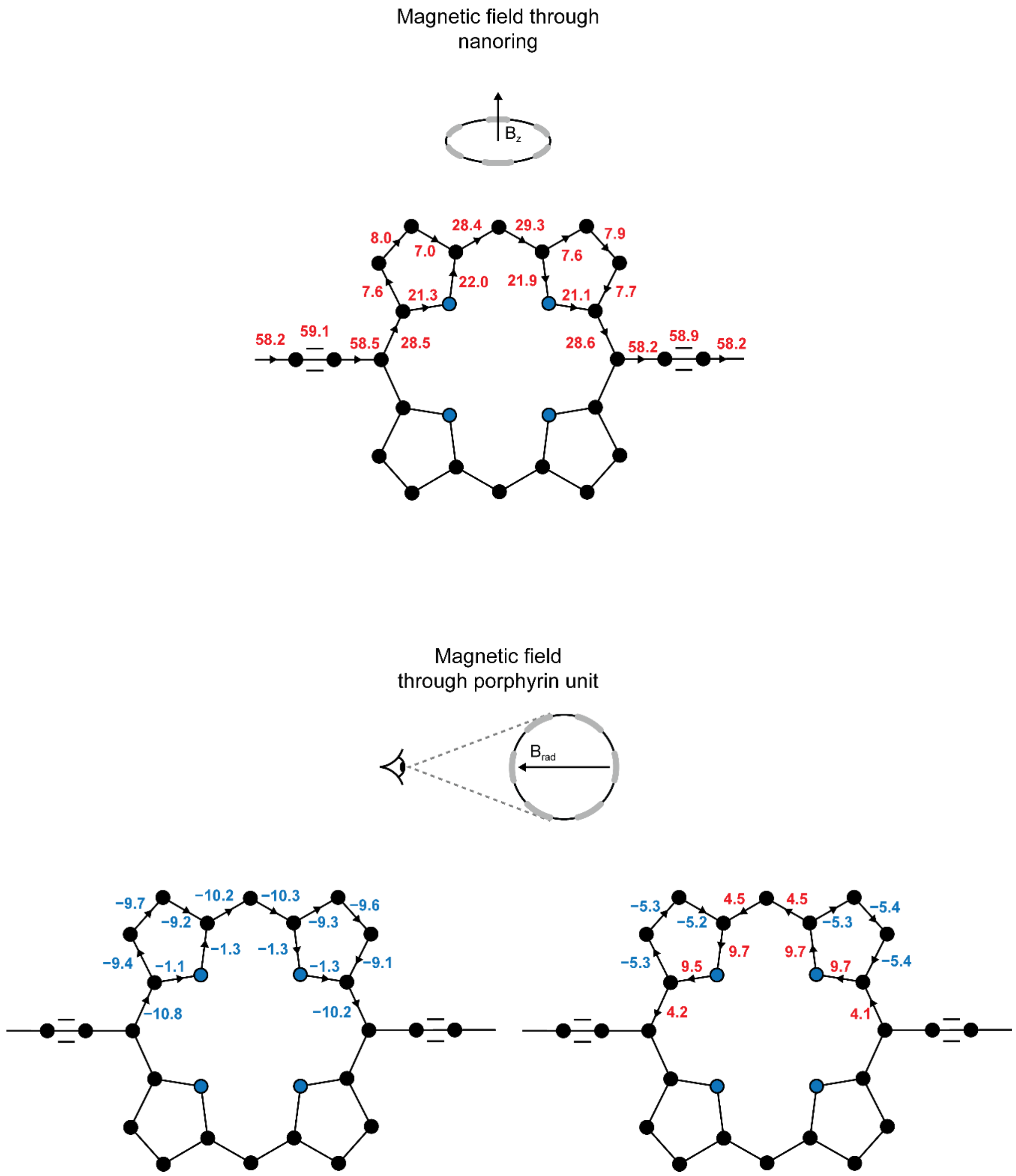

Figure S16: Ring current susceptibilities (in nA/T) calculated using GIMIC for one substituent porphyrin in ***c*-P6·T6**$^{4+}$. The upper figure shows global ring current susceptibilities through a substituent porphyrin. The lower figures show local ring current susceptibilities. Positive values represent paratropic ring currents, negative values represent diatropic ring currents.

In ***c*-P6·T6**$^{4+}$ the global ring current primarily flows through the inner pathway of each porphyrin, while 30% of the current takes the outer pathway.

As in ***c*-P6·T6**$^{2+}$, there are two porphyrin environments in ***c*-P6·T6**$^{4+}$. When the magnetic field is orientated perpendicular to a porphyrin, two porphyrins have paratropic ring currents and four porphyrins have diatropic ring currents. The diatropic currents appear to flow mostly through the outer pathway, which has a strength of 10 nA/T compared to 1 nA/T tracing the inner path. The two porphyrins with paratropic ring currents seem to have two "levels" of aromaticity: the porphyrin aromaticity, and the pyrrole aromaticity. In this case, the porphyrin is weakly antiaromatic and each pyrrole subunit aromatic. This has

the interesting effect of the porphyrin ring current being reinforced over the pyrrole $C_a – N – C_a$ bridge, hence the dark red color (Figure S15).

S5.4. Ring current pathways in *c*-P6·T6^{6+}

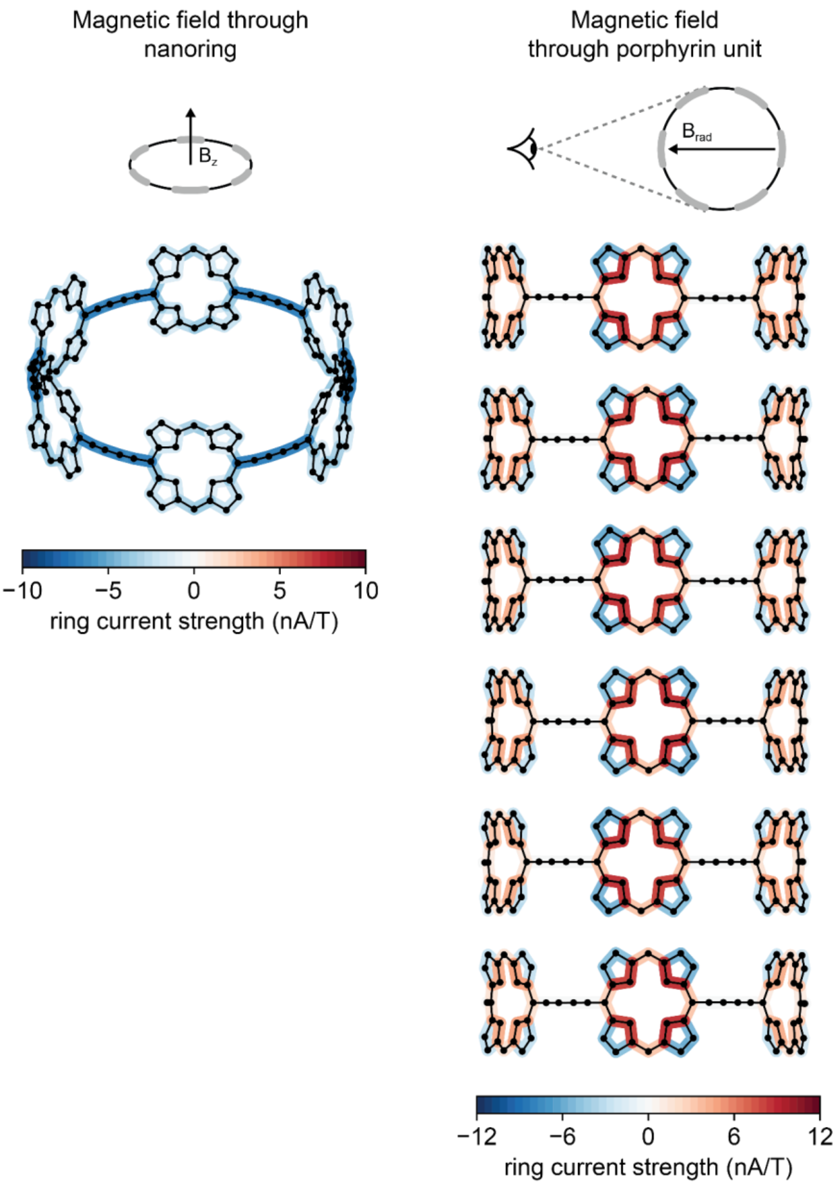

Figure S17: Local ring currents induced in ***c*-P6·T6^{6+}** by application of a magnetic field along a vector parallel to the radial axis of the nanoring (from the center of the nanoring to the center of a porphyrin), pointing towards the viewer in each subfigure. Each subfigure shows a different porphyrin subunit. Calculated using GIMIC (BLYP35/6-31G*).

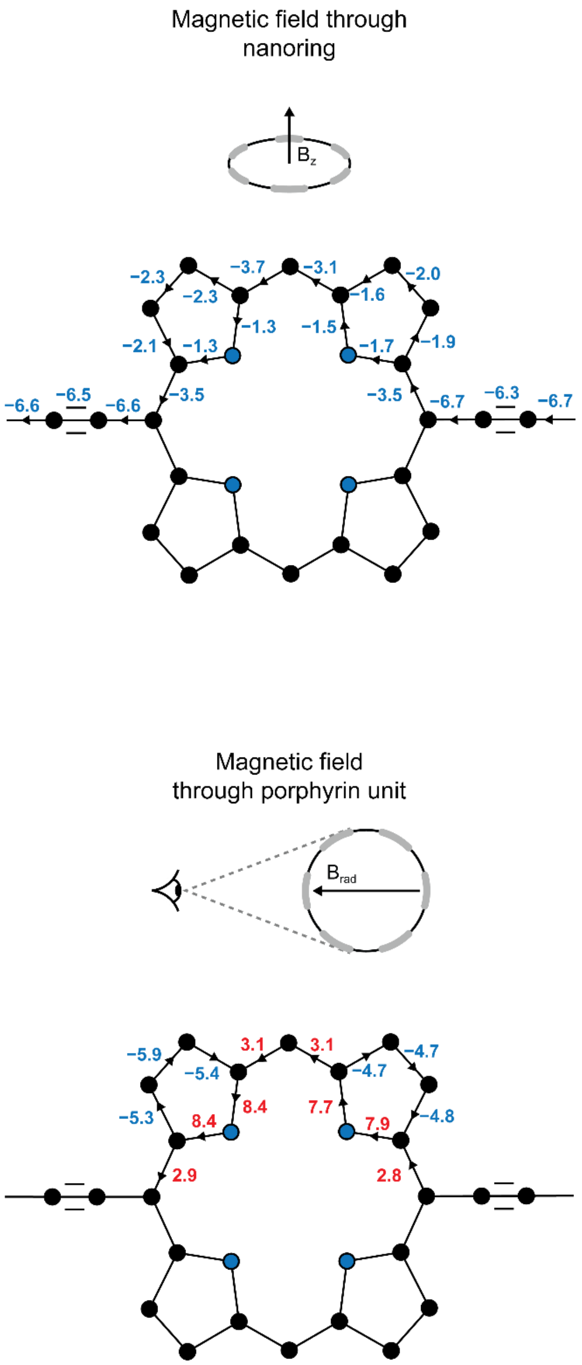

Figure S18: Ring current susceptibilities (in nA/T) calculated using GIMIC for one substituent porphyrin in ***c*-P6·T6**$^{6+}$. The upper figure shows global ring current susceptibilities through a substituent porphyrin. The lower figures show local ring current susceptibilities. Positive values represent paratropic ring currents, negative values represent diatropic ring currents.

***c*-P6·T6**$^{6+}$ sustains a weak global aromatic ring current. As with the previously discussed (anti)aromatic nanorings, the global ring current splits equally at the butadiyne-connected C_a – C_m bonds. As the current passes through the porphyrin, it splits equally at the C_a atom of each pyrrole, the same as it does in the globally-aromatic ***c*-P6·T6**$^{2+}$.

Unlike ***c*-P6·T6**$^{2+}$ and ***c*-P6·T6**$^{4+}$, the local ring currents of the six substituent porphyrins in ***c*-P6·T6**$^{6+}$ are equivalent. These ring currents appear to have the same structure as those in the antiaromatic subunits of ***c*-P6·T6**$^{4+}$: a local porphyrin antiaromaticity with aromatic pyrrole subunits.

S5.4. Ring current pathways in *c*-P6·T6^{12+}

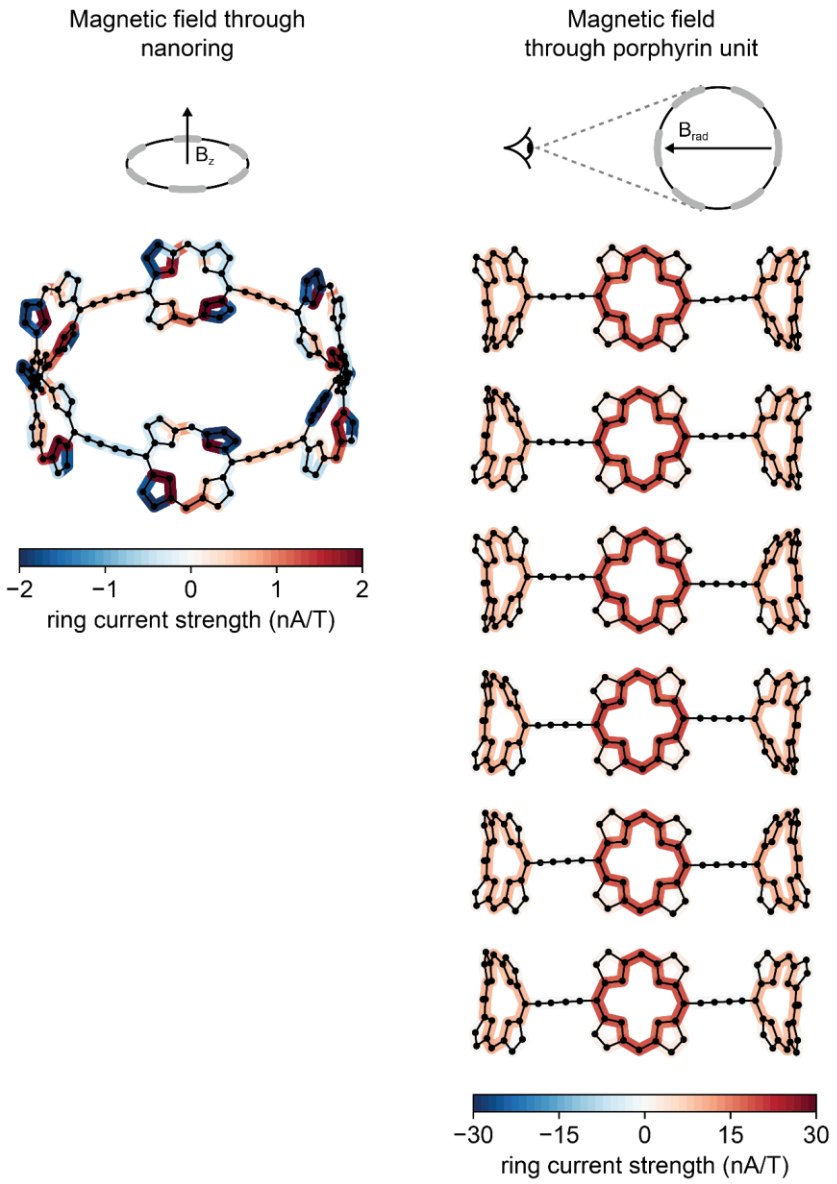

Figure S19: Local ring currents induced in ***c*-P6·T6^{12+}** by application of a magnetic field along a vector parallel to the radial axis of the nanoring (from the center of the nanoring to the center of a porphyrin), pointing towards the viewer in each subfigure. Each subfigure shows a different porphyrin subunit. Calculated using GIMIC (BLYP35/6-31G*).

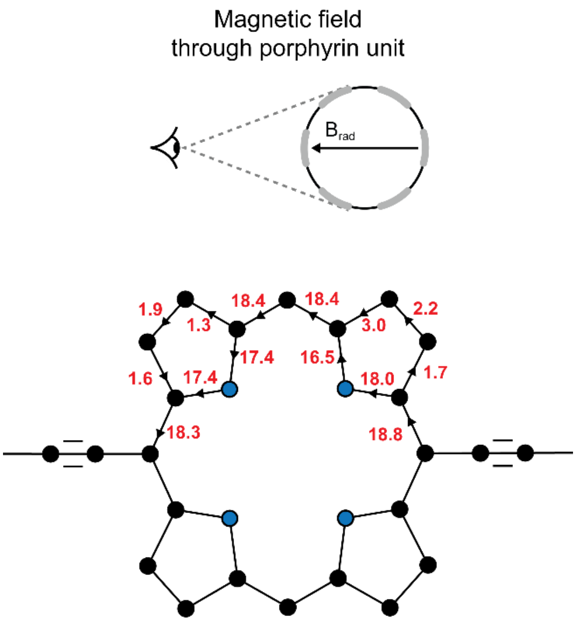

Figure S20: Ring current susceptibilities (in nA/T) calculated using GIMIC for one substituent porphyrin in ***c*-P6·T6**$^{6+}$, showing a local paratropic ring current.

While ***c*-P6·T6**$^{12+}$ does not sustain a global ring current, its warped geometry means some pyrrole subunits have weak ring currents when the magnetic field is applied perpendicular to the plane of the nanoring.

The six local porphyrin ring currents are equivalent, with 90% of its ring current travelling through the inner pathway (*i.e.* across the pyrrole N atom) and the remaining 10% through the outside of the pyrrole units.

S6. Supplementary figures and tables

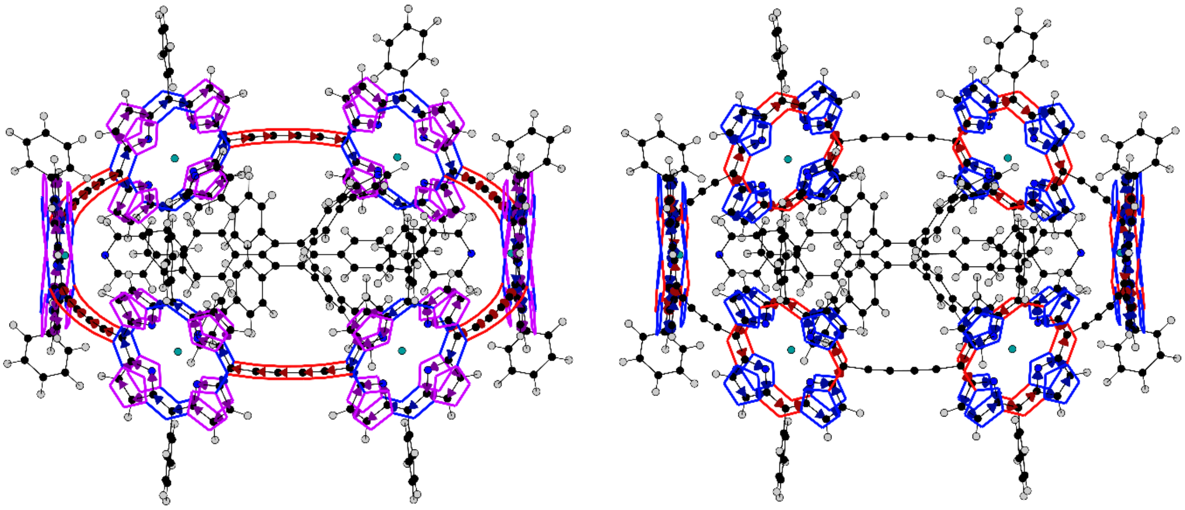

Figure S21: Global (left) and local (right) ring current models of ***c*-P6·T6**.

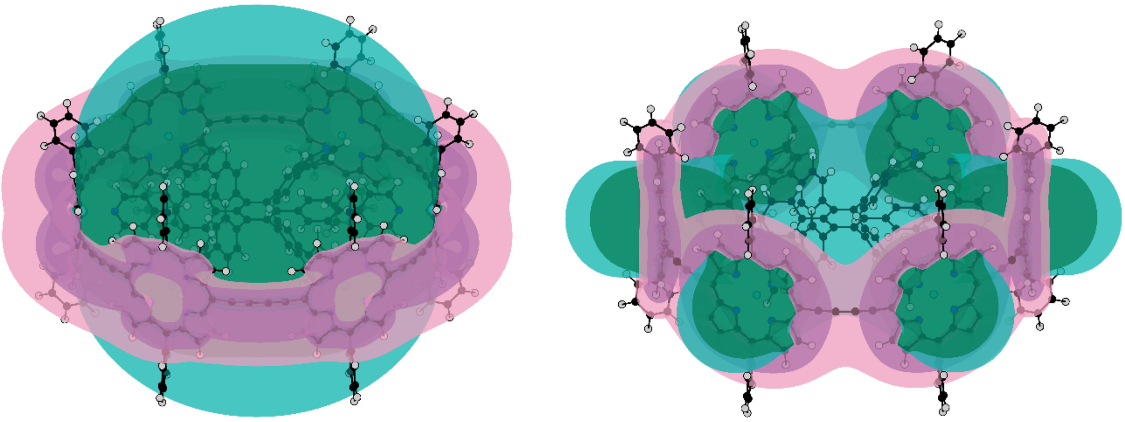

Figure S22: Induced isotropic shielding (*i.e.* RCGF values) in **c-P6·T6** for two different plausible ring current pathways. Left: a global ring current; right: local ring currents in each porphyrin (right). Pink and green denote different signs of the RCGF values.

Table S3: Experimental and DFT (BLYP35/6-31G*) ^{1}H NMR chemical shifts (in ppm) and Δδ values for ***c*-P6·T6**^{Q} (Q = 0, 2+, 4+, 6+, 12+), and local and global RCGFs derived from calculated structures for the same structures. Chemical shift values were taken from several sources, as indicated in the column labelled "Ref.".[8,9,14,15]

		***Q* = 0**		***Q* = 2+**		***Q* = 4+**		***Q* = 6+**		***Q* = 12+**		
		Exp.	DFT	Exp.	DFT	Exp.	DFT	Exp.	DFT	Exp.	DFT	Ref.
**Chemical shift (ppm)**	o$_{(in)}$	8.32	8.37	–	7.44	–	14.00	5.91	6.74	6.57	6.59	8
	o$_{(out)}$	8.06	7.93	–	8.00	–	5.45	7.78	7.92	7.31	8.12	8
	α	2.41	2.85	–	2.95	22.7	20.72	5.71	6.65	11.31	11.73	8, 9(**4+**)
	β	4.99	4.86	–	4.30	19.9	17.98	5.27	5.80	8.83	8.82	8, 9(**4+**)
	γ	5.45	5.52	2.69	4.90	–	16.76	5.17	5.94	7.94	8.35	8, 9(**2+**)
	δ	5.56	5.77	3.00	5.16	–	16.02	5.17	6.01	7.73	8.17	8, 9(**2+**)
	CH$_{2(in)}$	–	–	−0.25	–	4.73	–	0	–	–	–	14
	CH$_{2(out)}$	–	–	0.88	–	0.8	–	0.73	–	–	–	14
	m$_{(in)}$	–	7.92	–	7.5	–	10.83	–	7.32	–	7.48	
	m$_{(out)}$	–	7.86	–	7.78	–	7.86	–	7.68	–	7.67	
	CF$_3$	–	–	−64.7	–	–	–	−62.9	–	–	–	14
**Δδ values (ppm)**	o$_{(in)}$	0.7	0.93	–	0	–	6.56	−1.71	−0.7	−1.05	−0.85	8, 15
	o$_{(out)}$	0.44	0.48	–	0.56	–	−1.99	0.16	0.47	−0.31	0.68	8, 15
	α	−6.31	−5.79	–	−5.69	14.16	12.08	−2.83	−1.99	2.77	3.09	8
	β	−2.34	−2.31	–	−2.87	12.57	10.81	−2.06	−1.37	1.5	1.65	8
	γ	−1.79	−1.76	−4.55	−2.38	–	9.48	−2.07	−1.34	0.7	1.07	8, 15(**2+**)
	δ	−1.46	−1.44	−4.02	−2.05	–	8.81	−1.85	−1.20	0.71	0.96	8, 15(**2+**)
	CH$_{2(in)}$	–	–	−1.03	–	3.95	–	−0.78	–	–	–	14
	CH$_{2(out)}$	–	–	0.1	–	0.02	–	−0.05	–	–	–	14
	m$_{(in)}$	–	0.48	–	0.06	–	3.39	–	−0.12	–	0.04	
	m$_{(out)}$	–	0.42	–	0.34	–	0.42	–	0.24	–	0.22	
	CF$_3$	–	–	−4.4	–	–	–	−2.6	–	–	–	14
**RCGF$_{global}$ (μT/nA)**	o$_{(in)}$	0.1168		0.1131		0.1077		0.0996		0.0655		
	o$_{(out)}$	−0.0385		−0.0409		−0.0441		−0.0471		−0.0639		
	α	0.2434		0.2424		0.2409		0.2391		0.2340		
	β	0.1973		0.1969		0.1962		0.1953		0.1927		
	γ	0.1725		0.1726		0.1725		0.1724		0.1713		
	δ	0.1589		0.1590		0.1590		0.1588		0.1580		
	CH$_{2(in)}$	–		0.0435		0.0435		0.0435		–		
	CH$_{2(out)}$	–		−0.0134		−0.0134		−0.0134		–		
	m$_{(in)}$	0.0612		0.0585		0.0546		0.0491		0.0264		
	m$_{(out)}$	0.0047		0.0030		0.0007		−0.0018		−0.0149		
	CF$_3$	–		0.1735		–		0.1735		–		
**RCGF$_{local}$ (μT/nA)**	o$_{(in)}$	−0.0352		−0.0380		−0.0421		−0.0476		−0.0643		
	o$_{(out)}$	−0.0259		−0.0231		−0.0195		−0.0166		0.0155		
	α	0.2352		0.2328		0.2288		0.2230		0.1976		
	β	0.0983		0.0977		0.0966		0.0948		0.0879		
	γ	0.0703		0.0699		0.0692		0.0680		0.0632		
	δ	0.0587		0.0584		0.0579		0.0569		0.0531		
	CH$_{2(in)}$	–		−0.0107		−0.0107		−0.0107		–		
	CH$_{2(out)}$	–		0.0077		0.0077		0.0077		–		
	m$_{(in)}$	−0.0201		−0.0209		−0.0221		−0.0235		−0.0257		
	m$_{(out)}$	−0.0191		−0.0184		−0.0173		−0.0161		−0.0057		
	CF$_3$	–		0.0622		–		0.0622		–		

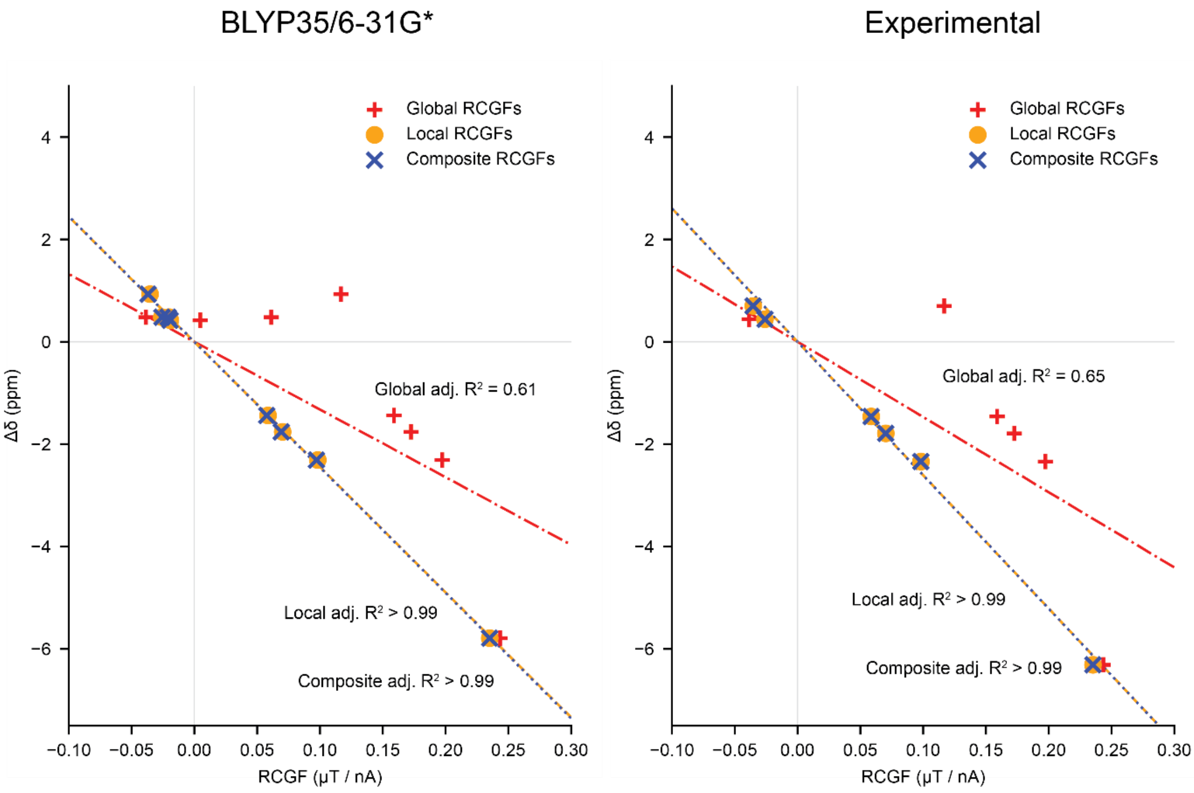

Figure S23: Comparison of single current loop models (local, global), and multiple-current loop models for neutral ***c*-P6·T6**, using NMR chemical shifts derived from DFT (left) and experiment (right). The RCGFs for the MCL model ("Composite RCGFs") are defined in section S1. Adjusted R^2 values account for the number of parameters in the model: MCL = 2, SCL = 1.

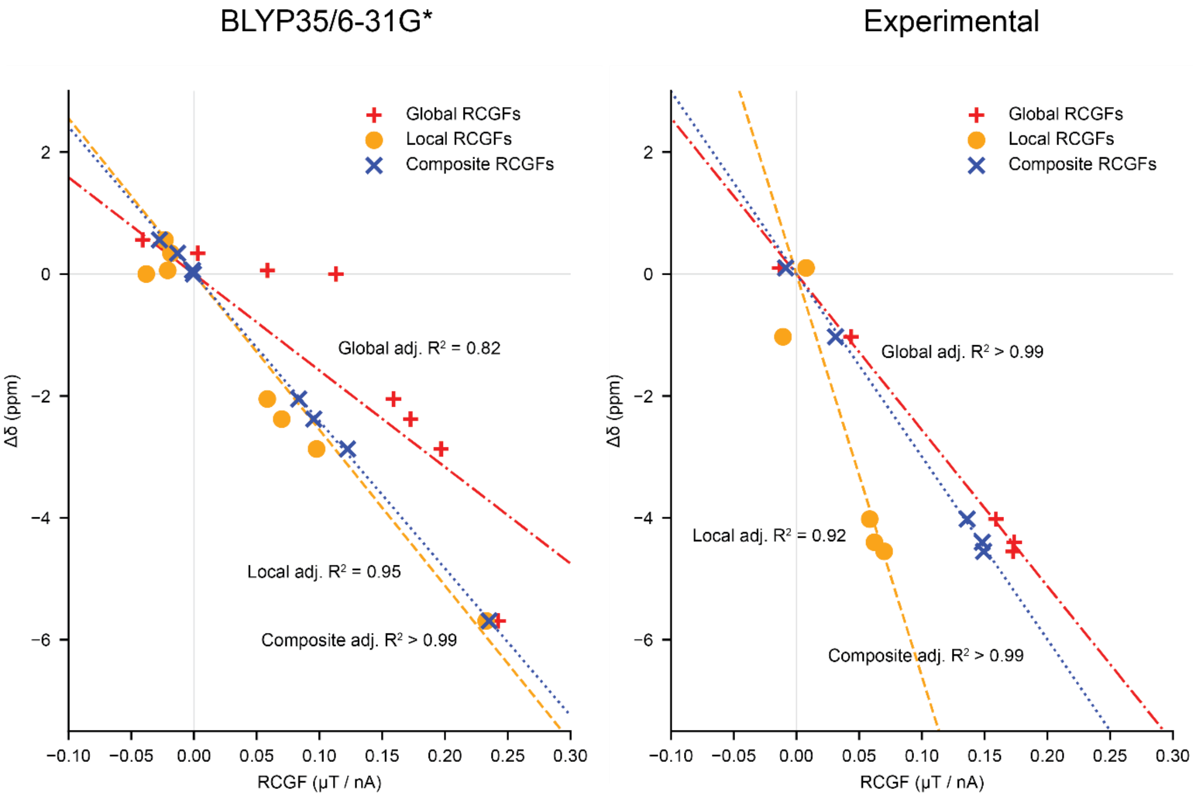

Figure S24: Comparison of single current loop models (local, global), and multiple-current loop models for ***c*-P6·T6**$^{2+}$, using NMR chemical shifts derived from DFT (left) and experiment (right). The RCGFs for the MCL model ("Composite RCGFs") are defined in section S1. Adjusted R^2 values account for the number of parameters in the model: MCL = 2, SCL = 1.

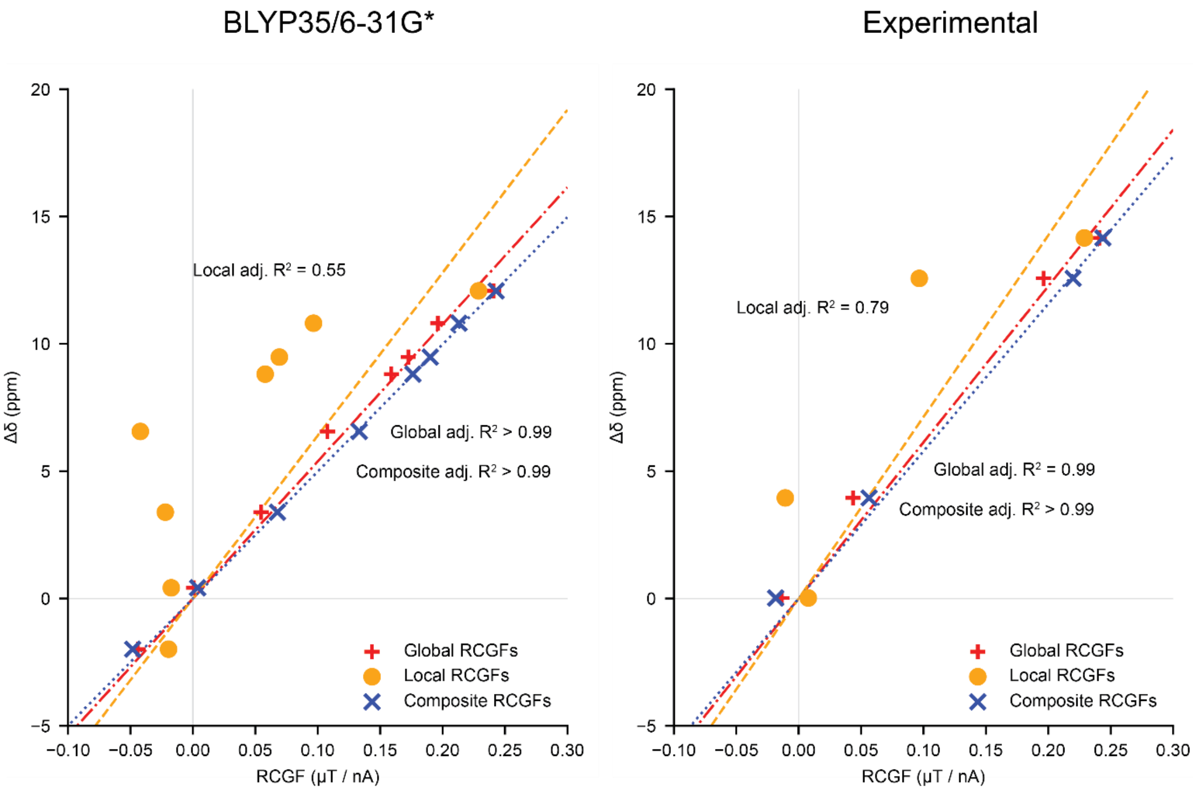

Figure S25: Comparison of single current loop models (local, global), and multiple-current loop models for ***c*-P6·T6**$^{4+}$, using NMR chemical shifts derived from DFT (left) and experiment (right). The RCGFs for the MCL model ("Composite RCGFs") are defined in section S1. Adjusted R^2 values account for the number of parameters in the model: MCL = 2, SCL = 1.

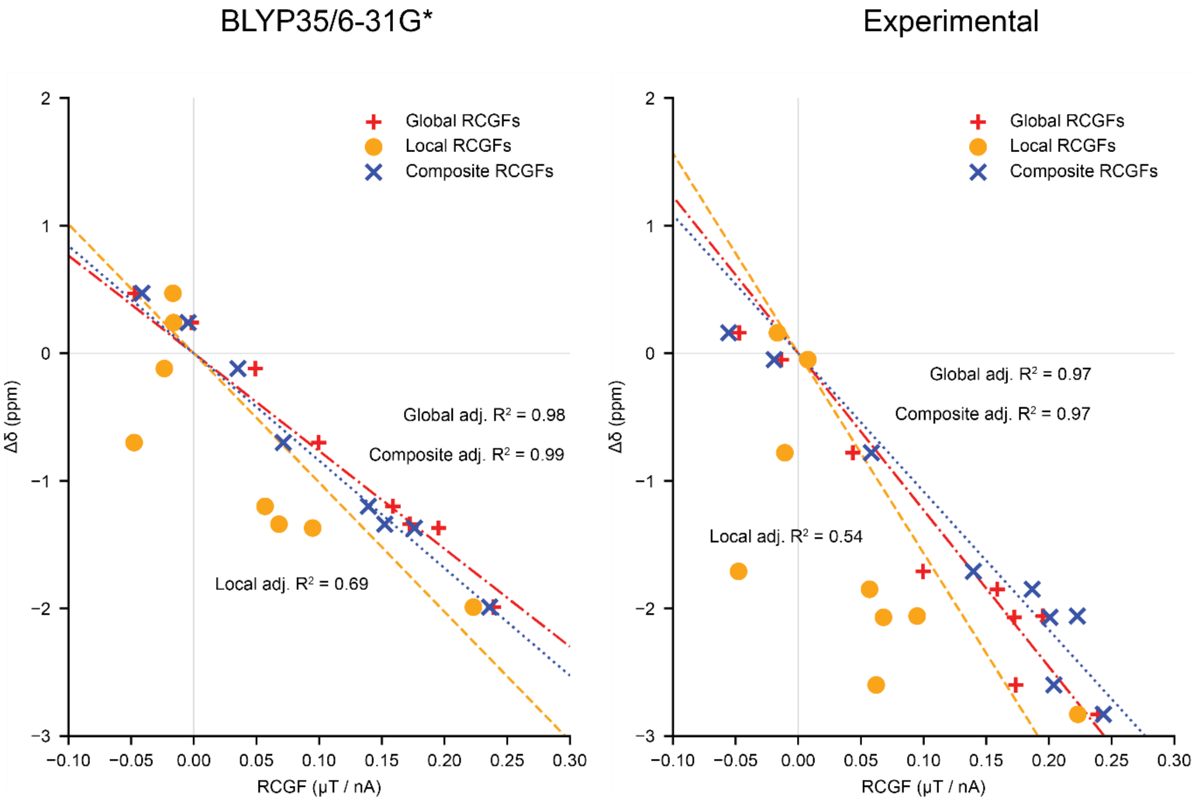

Figure S26: Comparison of single current loop models (local, global), and multiple-current loop models for ***c*-P6·T6**$^{6+}$, using NMR chemical shifts derived from DFT (left) and experiment (right). The RCGFs for the MCL model ("Composite RCGFs") are defined in section S1. Adjusted R^2 values account for the number of parameters in the model: MCL = 2, SCL = 1.

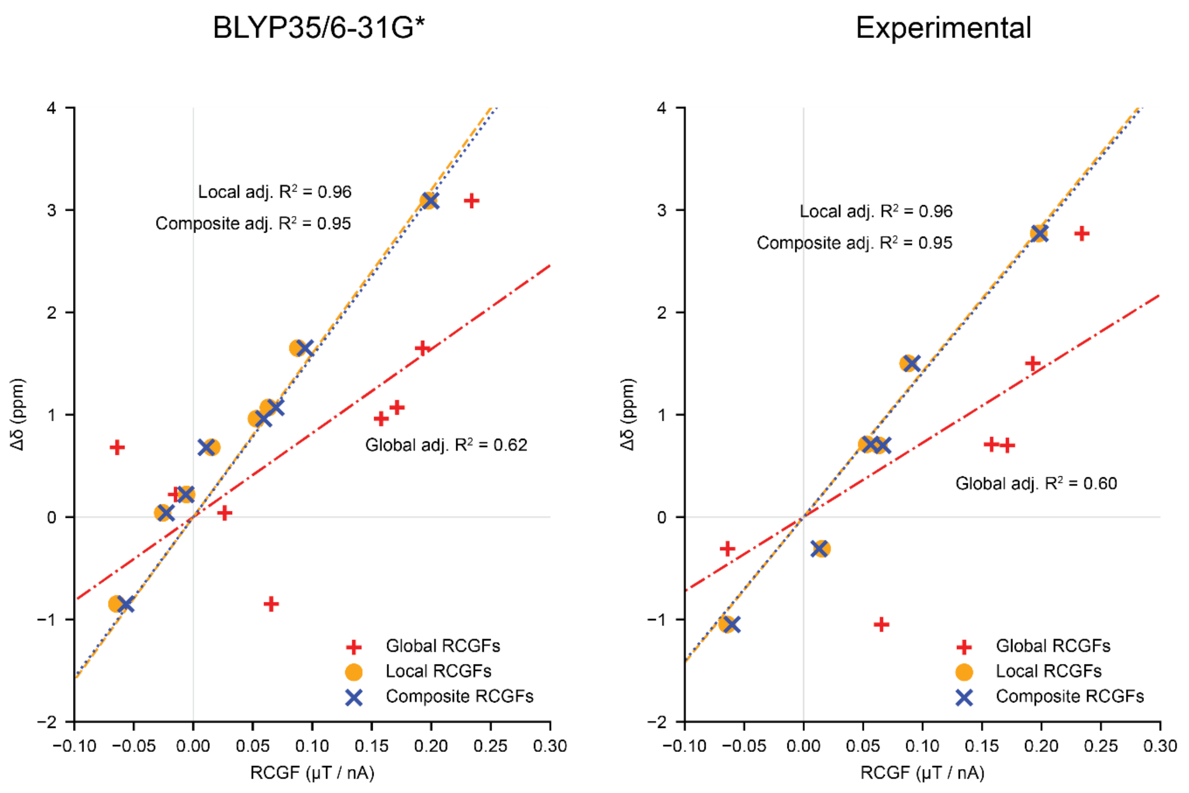

Figure S27: Comparison of single current loop models (local, global), and multiple-current loop models for ***c*-P6·T6**$^{12+}$, using NMR chemical shifts derived from DFT (BLYP35/6-31G*, left) and experiment (right). The RCGFs for the MCL model ("Composite RCGFs") are defined in section S1. Adjusted R^2 values account for the number of parameters in the model: MCL = 2, SCL = 1.

Table S4: Comparison of single current loop (SCL) model and multiple current loop (MCL) model fits for ***c*-P6·T6**^{Q} (where Q = 0, +2, +4, +6 and +12). The DFT results were calculated using BLYP35/6-31G*.

Q	**Method**	**SCL (nA/T)**		**MCL (nA/T)**		**RMSE (ppm)**		
		**Local**	**Global**	**Local**	**Global**	**SCL_{local}**	**SCL_{global}**	**MCL**
**0**	Expt.	−26.1	−14.7	−26.1	0.03	0.18	1.58	0.18
	DFT	−24.5	−13.2	−24.8	0.26	0.07	1.39	0.07
**+2**	Expt.	−66.0	−25.6	−6.85	−23.1	0.84	0.13	0.10
	DFT	−25.6	−15.8	−18.1	−6.00	0.51	1.01	0.06
**+4**	Expt.	71.6	61.4	−13.7	71.6	3.85	0.87	0.65
	DFT	64.0	53.8	−8.50	58.4	4.89	0.50	0.18
**+6**	Expt.	−15.7	−12.3	2.95	−13.8	1.17	0.31	0.27
	DFT	−10.1	−7.67	−1.61	−6.81	0.58	0.15	0.12
**+12**	Expt.	14.2	7.25	13.6	0.45	0.26	0.82	0.26
	DFT	16.0	8.20	14.8	0.92	0.27	0.80	0.26

In ***c*-P6·T6** and ***c*-P6·T6**$^{12+}$, the MCL model offers little improvement over the SCL_{local} model, on account of the lack of global ring current in these oxidation states. Using DFT and experimental data, the RMSE values are mostly the same between these two current loop models.

The MCL model offers more improvement in the +2, +4 and +6 oxidation states. Using experimental chemical shifts of ***c*-P6·T6**$^{2+}$, the MCL model offers only a small improvement over the fully global current loop model (0.10 vs 0.13 RMSE), however the difference is much larger with DFT chemical shifts (1.01 for SCL_{global} and 0.06 for MCL). The RMSEs of the MCL model for ***c*-P6·T6**$^{4+}$ are also much lower than that of the SCL models for both DFT and experimental data, suggesting multi-component shielding (i.e., local and global ring currents). Similarly for ***c*-P6·T6**$^{6+}$, the low RMSEs of the MCL model suggest the SCL model is inadequate in describing the true aromaticity of this oxidation state.

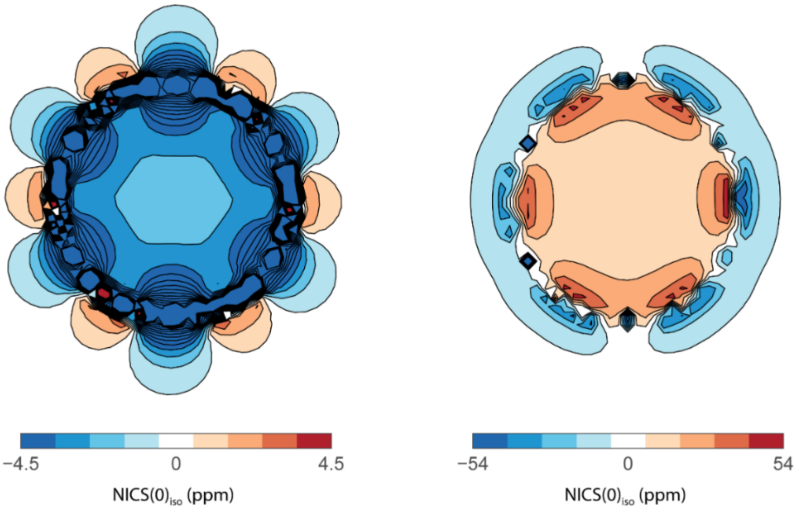

Figure S28: NICS plots of ***c*-P6^{2+}** (left) and ***c*-P6^{4+}** (right) calculated at the BLYP35/6-31G* level with a PCM using dichloromethane as solvent.

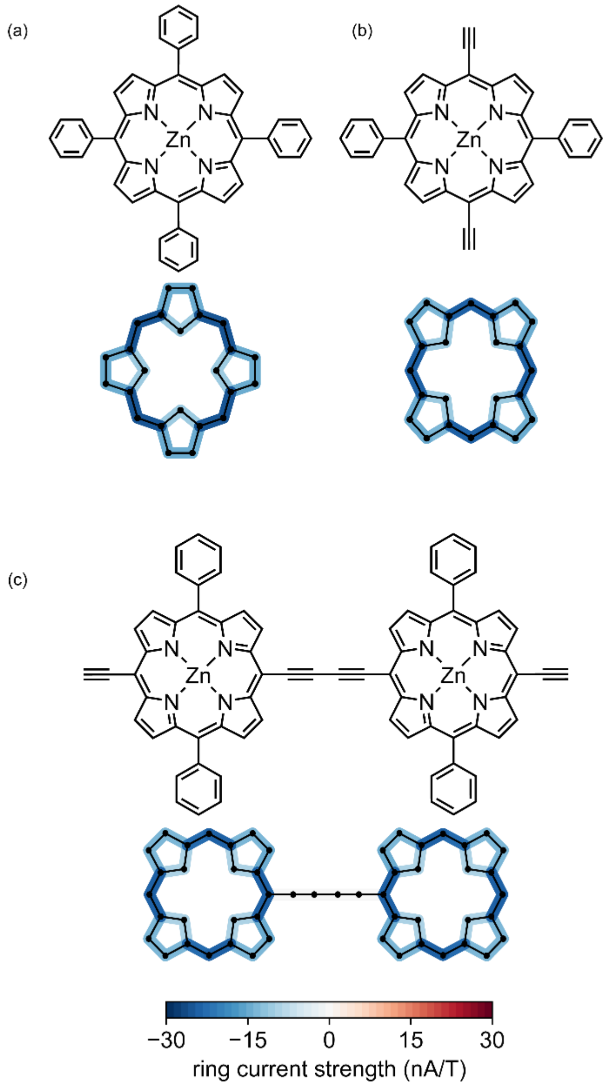

Figure S29: Ring current strengths calculated using GIMIC in (a) phenyl porphyrin, (b) phenyl diacetylene porphyrin and (c) phenyl diacetylene porphyrin dimer (right). Pendant aryl groups and acetylenes are omitted for clarity. BLYP35/6-31G*.

MCL analysis using the CAM-B3LYP functional

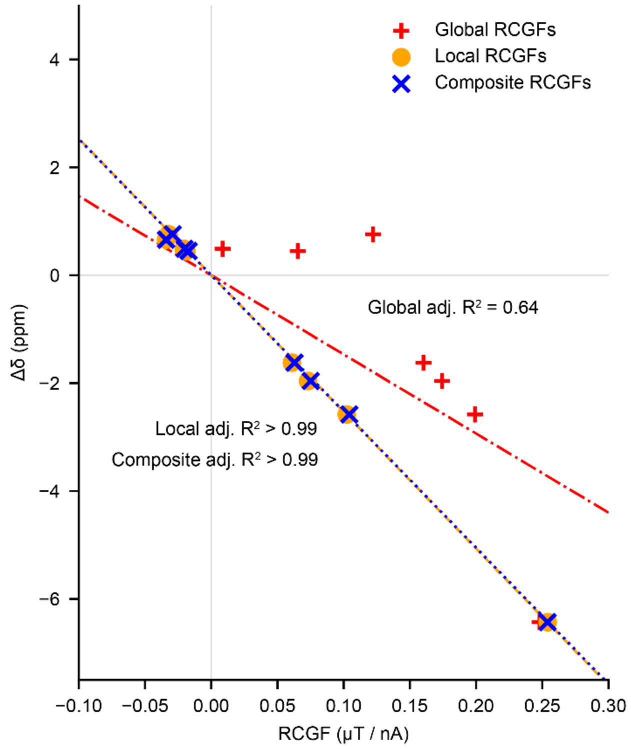

Figure S30: Comparison of single current loop models (local, global), and multiple-current loop models for ***c*-P6·T6**, using NMR chemical shifts derived from DFT (CAM-B3LYP/6-31G*). The RCGFs for the MCL model ("Composite RCGFs") are defined in section S1.

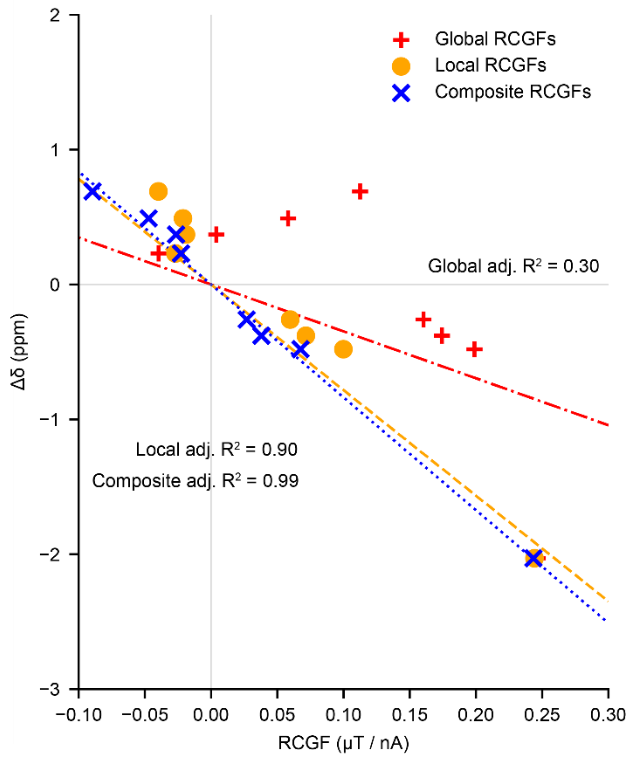

Figure S31: Comparison of single current loop models (local, global), and multiple-current loop models for ***c*-P6·T6^{4+}**, using NMR chemical shifts derived from DFT (CAM-B3LYP/6-31G*). This calculation used the geometry reported in ref. 16. The RCGFs for the MCL model ("Composite RCGFs") are defined in section S1.

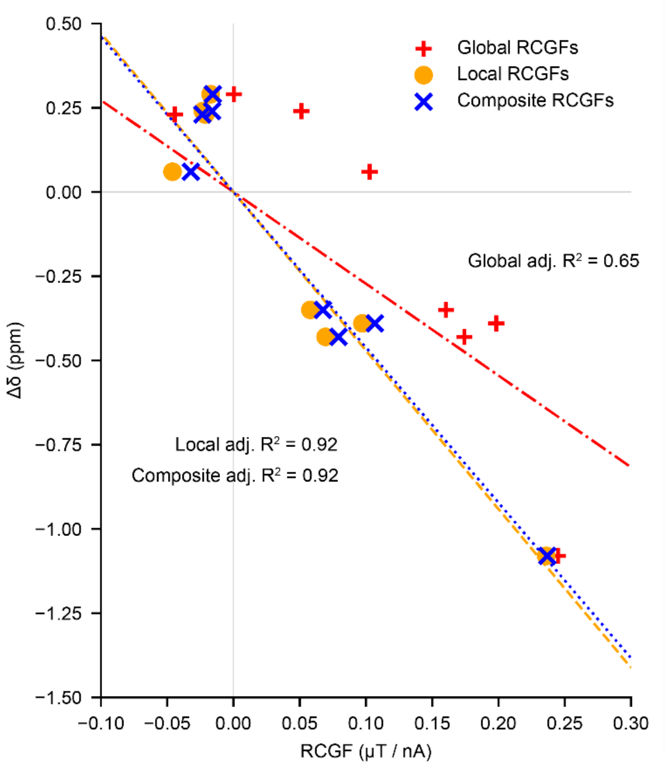

Figure S32: Comparison of single current loop models (local, global), and multiple-current loop models for ***c*-P6·T6**$^{6+}$, using NMR chemical shifts derived from DFT (CAM-B3LYP/6-31G*). The RCGFs for the MCL model ("Composite RCGFs") are defined in section S1.

Table S5: DFT (BLYP35 and CAM-B3LYP) ^{1}H NMR chemical shifts (in ppm) and Δδ values for ***c*-P6·T6^Q** (Q = 0, 4+, 6+), and calculated global and local ring current susceptibilities (nA/T). RCGFs calculated from CAM-B3LYP geometries were used in CAM-B3LYP MCL calculations, and the I/B values in the table are from the fit of DFT chemical shift differences using the MCL model. The basis set was 6-31G*.

		***Q* = 0**		***Q* = 4+**		***Q* = 6+**	
		CAM-B3LYP	BLYP35	CAM-B3LYP	BLYP35	CAM-B3LYP	BLYP35
**Chemical shift (ppm)**	$o_{(in)}$	8.23	8.37	8.15	14.00	7.52	6.74
	$o_{(out)}$	8.12	7.93	7.69	5.45	7.69	7.92
	α	2.25	2.85	6.65	20.72	7.60	6.65
	β	4.67	4.86	6.78	17.98	6.86	5.80
	γ	5.32	5.52	6.90	16.76	6.85	5.94
	δ	5.55	5.77	6.91	16.02	6.82	6.01
	$m_{(in)}$	7.91	7.92	7.95	10.83	7.70	7.32
	$m_{(out)}$	7.95	7.86	7.83	7.86	7.75	7.68
**Δδ values (ppm)**	$o_{(in)}$	0.76	0.93	0.69	6.56	0.06	−0.7
	$o_{(out)}$	0.66	0.48	0.23	−1.99	0.23	0.47
	α	−6.43	−5.79	−2.03	12.08	−1.08	−1.99
	β	−2.58	−2.31	−0.48	10.81	−0.39	−1.37
	γ	−1.96	−1.76	−0.38	9.48	−0.43	−1.34
	δ	−1.62	−1.44	−0.26	8.81	−0.35	−1.20
	$m_{(in)}$	0.45	0.48	0.49	3.39	0.24	−0.12
	$m_{(out)}$	0.49	0.42	0.37	0.42	0.29	0.24
**$(I/B)_{global}$ (nA/T)**		−0.5	−0.3	2.7	58.4	−0.4	−6.8
**$(I/B)_{local}$ (nA/T)**		−24.7	−24.8	−11.1	−8.5	−4.2	−1.6
**$RCGF_{global}$ (μT/nA)**	$o_{(in)}$	0.1222	0.1168	0.1125	0.1077	0.1028	0.0996
	$o_{(out)}$	−0.0326	−0.0385	−0.0396	−0.0441	−0.0443	−0.0471
	α	0.2480	0.2434	0.2466	0.2409	0.2449	0.2391
	β	0.1992	0.1973	0.1989	0.1962	0.1983	0.1953
	γ	0.1743	0.1725	0.1743	0.1725	0.1743	0.1724
	δ	0.1603	0.1589	0.1603	0.1590	0.1603	0.1588
	$m_{(in)}$	0.0653	0.0612	0.0582	0.0546	0.0513	0.0491
	$m_{(out)}$	0.0086	0.0047	0.0040	0.0007	0.0004	−0.0018
**$RCGF_{local}$ (μT/nA)**	$o_{(in)}$	−0.0322	−0.0352	−0.0398	−0.0421	−0.0459	−0.0476
	$o_{(out)}$	−0.0341	−0.0259	−0.0269	−0.0195	−0.0213	−0.0166
	α	0.2540	0.2352	0.2442	0.2288	0.2360	0.2230
	β	0.1023	0.0983	0.0999	0.0966	0.0973	0.0948
	γ	0.0731	0.0703	0.0715	0.0692	0.0695	0.0680
	δ	0.0609	0.0587	0.0597	0.0579	0.0581	0.0569
	$m_{(in)}$	−0.0188	−0.0201	−0.0212	−0.0221	−0.023	−0.0235
	$m_{(out)}$	−0.0208	−0.0191	−0.019	−0.0173	−0.0172	−0.0161

[34]Octaphyrin

The two ring current models for the [34]octaphyrin were based on the two viable conjugated paths. The RCM paths were split into two, above and below the normal plane (plane defined by the local atoms).

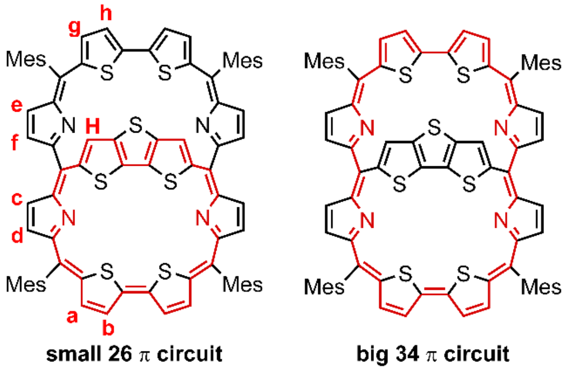

Figure S33: Structure of bicyclic DTT-bridged [34]octaphyrin with highlighted possible conjugated paths.

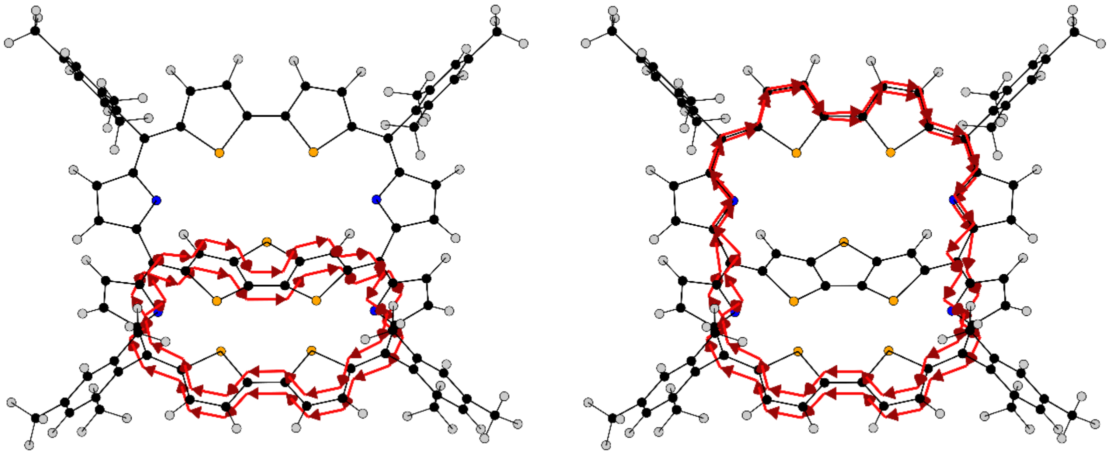

Figure S34: Ring current models of bicyclic DTT-bridged [34]octaphyrin with possible conjugated paths highlighted.

Table S6: Experimental and DFT (BLYP35/6-31G*) ^{1}H NMR chemical shifts (in ppm) and Δδ values for DTT-bridged [34]octaphyrin. As the non-aromatic reference for all experimental NMR data was used the default value of 5.8 ppm.

		**DTT-bridged [34]octapyrin**	
		Exp.	DFT
**Chemical shift (ppm)**	a	10.41	11.31
	b	12.08	12.55
	c	8.89	10.24
	d	10.27	9.29
	e	9.97	8.51
	f	8.25	9.91
	g	9.76	8.73
	h	8.49	9.55
	H	7.12	7.21
**Δδ values (ppm)**	a	4.61	5.44
	b	6.28	6.68
	c	3.09	4.37
	d	4.47	3.42
	e	4.17	2.64
	f	2.45	4.04
	g	3.96	2.86
	h	2.69	3.68
	H	1.32	1.34
**$RCGF_{global}$ (μT/nA)**	a	−0.1831	
	b	−0.2052	
	c	−0.1183	
	d	−0.0906	
	e	−0.1092	
	f	−0.1375	
	g	−0.1807	
	h	−0.2046	
	H	0.2842	
**$RCGF_{local}$ (μT/nA)**	a	−0.1721	
	b	−0.1936	
	c	−0.0745	
	d	−0.0824	
	e	−0.0215	
	f	−0.0617	
	g	−0.0012	
	h	0.0016	
	H	−0.1665	

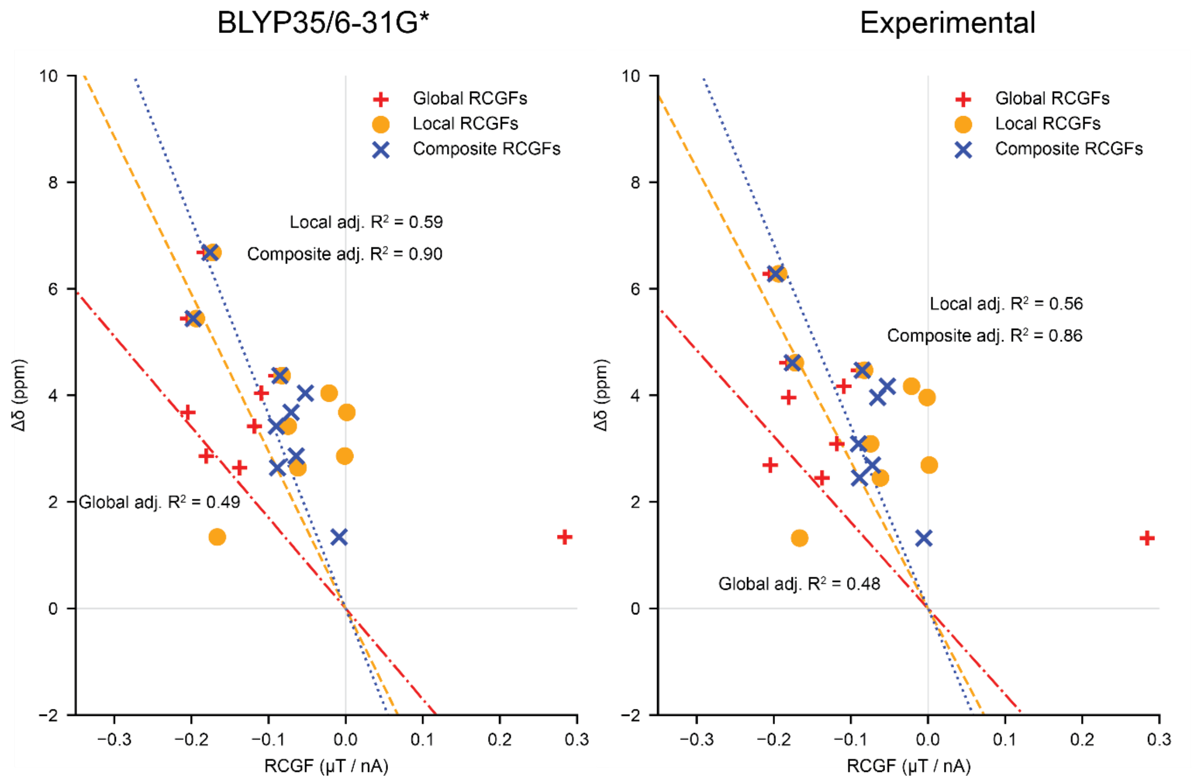

Figure S35: Comparison of single current loop models (local, global), and multiple-current loop models for bicyclic [34]octaphyrin using NMR chemical shifts derived from DFT (left) and experiment (right). The RCGFs for the MCL model ("Composite RCGFs") are defined in section S1. Adjusted R2 values account for the number of variables in the model: MCL = 2, SCL = 1.

Table 7: Comparison of single current loop (SCL) model and multiple current loop (MCL) model fits for DTT-bridged [34]octaphyrin.

Method	SCL (nA/T)		MCL (nA/T)		RMSE (ppm)		
	Local	Global	Local	Global	SCL_{local}	SCL_{global}	MCL
**Expt.**	−27.5	−16.2	−12.2	−22.0	2.44	2.67	1.29
**DFT**	−29.5	−17.0	−23.7	−12.8	2.47	2.78	1.17

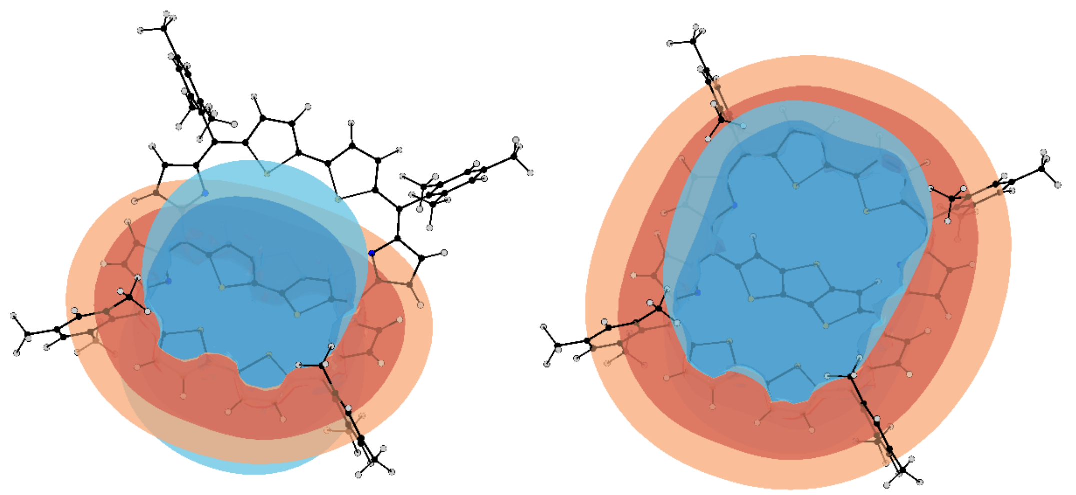

Figure S36: Calculated induced shielding in DTT-bridged [34]octaphyrin arising from the local and global ring currents.

Table S8: Reported [ref 10] and calculated (this work) chemical shifts at the BLYP35/6-31G* (with PCM, tetrahydrofuran) and B3LYP/def2-TZVP (no solvent model).

	Reported chemical shift (ppm)	BLYP35 chemical shift (ppm)	B3LYP chemical shift (ppm)
**a**	10.41	11.31	12.37
**b**	12.08	12.55	13.29
**c**	8.89	10.24	11.87
**d**	10.27	9.29	10.76
**e**	9.97	8.51	10.19
**f**	8.25	9.91	11.40
**g**	9.76	8.73	10.64
**h**	8.49	9.55	11.34
**H**	7.12	7.21	4.43

S7. Statistical analyses

We used the adjusted R^2 and corrected Akaike information criterion (AICc) to evaluate the goodness of fit for different current-loop models. These statistical methods evaluate the fit between a model and data, with a penalty for additional unnecessary parameters (*i.e.* they reward parsimony). These statistics can be used in conjunction with external knowledge (*e.g.* that there may be multiple ring current pathways in a molecule) to select the most appropriate model(s).

Since we have a relatively small number of chemical shifts for each species (n in Tables S9 and S10), the statistics cannot be relied upon in isolation. Addition of extra observations would have a large effect on both AIC and R^2 adj. For our present purposes we consider that a ΔAIC of >5 or a Δ-adj-R^2 of >0.01 ought to raise appreciable doubts about the statistical suitability of any model in a pairwise comparison.

Table S9 contains adjusted R^2 values for single current loops with and without an intercept term. In this table, higher values (closer to 1) suggest a better fit. It is notable that most of the fits are fairly good, except where the model does not describe the dominant ring current at all (*e.g.* a global SCL model for neutral ***c*-P6·T6**, for which adj-R^2 = 0.650, *cf.* 0.994 for the MCL model).

Table S10 contains AICc values for both SCL and MCL models. AICc values cannot be evaluated in isolation, but in a pairwise comparison the model with the lower AICc is generally the better model. For example, turning again to the neutral ***c*-P6·T6**, the global SCL model has a much higher AICc (25.5) than the local SCL model (AICc = –0.6), suggesting that the local SCL model is superior to the global SCL model. The MCL model (AICc = 4.4) is also clearly better than a global SCL model, but somewhat worse than a local-only model (this result is supported by the result in main text Fig 2, which shows that there is effectively no global ring current contribution in this molecule).

Taken together, the fit statistics do not provide a strong argument against the MCL model, or for/against the addition of an intercept parameter. In many cases the MCL model provides a similar or superior improvement to the SCL_{global} fit as adding an intercept term to the latter model. The inclusion of an intercept term in the MCL model is not justified because it offers only a minor (and inconsistent) improvement to the fit, while delivering no extra chemical insight. Its inclusion would risk over-parameterizing the system, especially where n is low. Generally, the addition of an intercept term has a negligible effect on the fitted values for the ring current susceptibilities. The fit could be improved, in the absence of an intercept term, by extending the MCL model to define more ring current cycles, or by correcting the Δδ values to remove contributions not attributable to ring current effects.

Table S9:: Adjusted R^2 for the SCL (either local or global) and MCL models, with and without a fitted intercept term. Fit parameters were calculated using the `statsmodels` package in Python.

	Species	Model / adjusted R^2						
		n	~$RCGF_{local}$	~$RCGF_{local}$ + c	~$RCGF_{global}$	~$RCGF_{global}$ + c	~$RCGF_{local}$ + $RCGF_{global}$	~$RCGF_{local}$ + $RCGF_{global}$ + c
c-P6·T6	Expt.	6	0.996	0.993	0.650	0.485	0.994	0.994
	DFT	8	0.999	0.999	0.613	0.595	0.999	1.000
c-P6·T6^{2+}	Expt.	5	0.923	0.826	0.998	0.995	0.999	0.999
	DFT	8	0.954	0.974	0.816	0.768	0.999	0.999
c-P6·T6^{4+}	Expt.	4	0.788	0.634	0.989	0.993	0.991	1.000
	DFT	8	0.553	0.520	0.995	0.993	0.999	0.999
c-P6·T6^{6+}	Expt.	9	0.543	0.404	0.967	0.943	0.971	0.944
	DFT	8	0.689	0.643	0.980	0.986	0.985	0.987
c-P6·T6^{12+}	Expt.	6	0.960	0.961	0.604	0.528	0.951	0.983
	DFT	8	0.956	0.985	0.624	0.392	0.953	0.990
[34]-	Expt.	9	0.563	–0.061	0.478	0.312	0.862	0.536
octaphyrin	DFT	9	0.594	–0.002	0.485	0.317	0.896	0.672

Table S10: Akaike Information Criterion (corrected, AICc) for the SCL and MCL models, with and without a fitted intercept term. Lower values are better, and values should be compared pairwise. Fit parameters were calculated using the `statsmodels` package in Python.

	Species	Model / AICc						
		n	~$RCGF_{local}$	~$RCGF_{local}$ + c	~$RCGF_{global}$	~$RCGF_{global}$ + c	~$RCGF_{local}$ + $RCGF_{global}$	~$RCGF_{local}$ + $RCGF_{global}$ + c
c-P6·T6	Expt.	6	–0.6	3.6	25.5	29.7	4.4	11.7
	DFT	8	–16.5	–13.3	30.6	32.5	–13.5	–17.6
c-P6·T6^{2+}	Expt.	5	15.8	20.6	–2.7	2.6	0.7	13.8
	DFT	8	14.5	10.1	25.2	27.5	–16.5	–12.1
c-P6·T6^{4+}	Expt.	4	n.d.	n.d.	n.d.	n.d.	n.d.	n.d.
	DFT	8	50.8	47.0	14.4	12.9	1.9	–2.9
c-P6·T6^{6+}	Expt.	9	30.9	25.8	7.1	4.8	8.2	7.9
	DFT	8	16.6	16.4	–5.2	–9.8	–5.0	–6.1
c-P6·T6^{12+}	Expt.	6	3.9	6.6	17.6	21.6	8.7	10.0
	DFT	8	4.6	–4.2	21.8	25.4	7.7	–3.6
[34]-	Expt.	9	44.2	36.6	45.8	32.7	36.1	32.5
octaphyrin	DFT	9	44.4	37.5	46.5	34.0	34.3	30.8

S8. References

1 S. Grimme, J. Antony, S. Ehrlich and H. Krieg, *J. Chem. Phys.*, 2010, **132**, 154104.
2 R. Ditchfield, W. J. Hehre and J. A. Pople, *J. Chem. Phys.*, 1971, **54**, 724–728.
3 W. J. Hehre, R. Ditchfield and J. A. Pople, *J. Chem. Phys.*, 1972, **56**, 2257–2261.
4 P. C. Hariharan and J. A. Pople, *Theor. Chim. Acta*, 1973, **28**, 213–222.
5 V. A. Rassolov, J. A. Pople, M. A. Ratner and T. L. Windus, *J. Chem. Phys.*, 1998, **109**, 1223–1229.
6 T. Yanai, D. P. Tew and N. C. Handy, *Chem. Phys. Lett.*, 2004, **393**, 51–57.
7 M. Renz, K. Theilacker, C. Lambert and M. Kaupp, *J. Am. Chem. Soc.*, 2009, **131**, 16292–16302.
8 M. D. Peeks, T. D. W. Claridge and H. L. Anderson, *Nature*, 2017, **541**, 200–203.
9 M. Rickhaus, M. Jirasek, L. Tejerina, et al., *Nat. Chem.*, 2020, **12**, 236–241.
10 W.-Y. Cha, T. Kim, A. Ghosh, et al., *Nat. Chem.*, 2017, **9**, 1243–1248.
11 J. Jusélius, D. Sundholm and J. Gauss, *J. Chem. Phys.*, 2004, **121**, 3952–3963.
12 S. Taubert, D. Sundholm and J. Jusélius, *J. Chem. Phys.*, 2011, **134**, 054123.
13 H. Fliegl, S. Taubert, O. Lehtonen and D. Sundholm, *Phys. Chem. Chem. Phys.*, 2011, **13**, 20500–20518.
14 M. Jirásek, H. L. Anderson and M. D. Peeks, *Acc. Chem. Res.*, 2021, **54**, 3241–3251.
15 J.-R. Deng, D. Bradley, M. Jirásek, H. L. Anderson and M. D. Peeks, *Angew. Chem. Int. Ed.*, 2022, **61**, e202201231.
16 I. Casademont-Reig, R. Guerrero-Avilés, E. Ramos-Cordoba, M. Torrent-Sucarrat and E. Matito, *Angew. Chem. Int. Ed.*, 2021, **60**, 24080–24088.
